# Synthesis and characterization of polyurethane flexible foams provided from PET derivatives, fly ash, and glass wastes^☆^

**DOI:** 10.1016/j.heliyon.2023.e23097

**Published:** 2023-12-01

**Authors:** Adriana Cornelia Mârșolea (Cristea), Alexandra Mocanu, Paul Octavian Stănescu, Oana Brincoveanu, Cristina Orbeci, Roberta Irodia, Cristian Pîrvu, Adrian Dinescu, Constantin Bobirica, Edina Rusen

**Affiliations:** a University Politehnica of Bucharest, Faculty of Chemical Engineering and Biotechnologies, 1- 7 Gh. Polizu Street, 011061 Bucharest, Romania; b National Institute for Research and Development in Microtechnologies – IMT Bucharest, 126A Erou Iancu Nicolae Street, 077190 Bucharest, Romania; c Advanced Polymer Materials Group, Faculty of Chemical Engineering and Biotechnology, University Politehnica of Bucharest, 1-7 Gh. Polizu Street, 011061 Bucharest, Romania; d Research Institute of the University of Bucharest, ICUB Bucharest, Soseaua Panduri, nr. 90, Sector 5, 050663, Bucureşti, Romania

**Keywords:** PET waste, Depolymerization, fly ash, Glass waste, Polyurethanes

## Abstract

The aim of this study involved the synthesis and characterization of polyurethane (PUR) foams obtained from poly(ethylene terephthalate) (PET) depolymerization products and two types of filling agents, namely fly ash and glass waste. The depolymerized PET-based products were obtained by zinc acetate-catalyzed glycolysis process in diethylene glycol (DEG) as a co-reactant. The resulting glycolysis products were contacted with methylene diphenyl diisocyanate, castor oil, and reinforcing agents. The resulting PUR specimens were characterized by Fourier transform infrared spectroscopy (FT-IR), scanning electron microscopy (SEM), energy dispersive X-ray analysis (EDX), EDX mapping, mechanical tests, and thermal analysis. The analysis confirmed that the best mechanical performances were registered by the specimens with the lowest concentration of filling agent, while the highest thermal resistance was achieved by the PUR foams with the highest concentration of reinforcing agent.

## 1 Introduction

The polycondensation reaction of dimethyl terephthalate and ethylene glycol produces polyethylene terephthalate (PET), a saturated polyester that finds application in a wide range of industries. Based on its semi-crystallinity, transparency, mechanical properties, and gas-barrier characteristics, PET found its use in the packaging industry as bottles for food beverages, in the textile industry as fibers, geotextiles, or technical textiles, in the coatings industry as film-forming material, and in the automotive industry as airbags, seat belts or electronic devices [1,2].

During the pandemic of COVID 19, many consumers switched to online shopping which created a massive demand for packaging in many commercial sectors that finally led to a global PET market size of $44.30 billion in 2022 [3,4] Although there is a continuous demand for PET-based products for reprocessing PET waste or other fossil-based plastics, there is still a gap in the loop of the recycling process that must be REACH compliant according to the European Union requirements [3].

In the recycling policy for PET waste, 4 different categories have so far identified in which re-extrusion is classified as primary recovery, mechanical recycling as secondary recycling, chemical recycling as tertiary recycling, and energy recovery as quaternary recycling [5].

A good opportunity to give PET waste a new life determined many researchers to develop new chemical strategies that include mainly: (i) hydrolysis; (ii) glycolysis; (iii) methanolysis; (iv) depolymerization methods, and (v) ammonolysis [5].

Previously, our team was involved in PET recycling by the chemical method through glycolysis reactions both in conventional [6] and microwave-assisted reactions [7]. To produce new innovative composite materials our research was continuously directed to the reuse of oligomers, respectively monomers resulting from the depolymerization process of PET. Thus, the first innovative products that we obtained were polyurethane adhesives and rigid polyurethane foams [8].

Polyurethanes (PUR) are a special family of materials with a variety of uses, including coatings, adhesives, elastomers, sealants, films, flexible, semi-rigid, and rigid foams [9]. Polyurethane foams are the most versatile products, being highly used in different applications depending on their morphology and mechanical properties as flexible, semi-rigid, or rigid foams [9,10]. PUR foam flexible products account for up to 65 % of the foam market being mostly used as mattresses in furniture industry, while the rigid version is used mostly as thermal insulating material in construction field [11].

The composition of the polyols and isocyanate precursors, as well as the distribution of the soft and hard segments of PURs, have a significant impact on the mechanical and functional qualities of the final foam that results from the interaction of diols and diisocyanates to produce PURs [12,13]. PURs foams have a number of significant qualities that make them ideal for thermal isolation in the civil engineering sector, these qualities including outstanding mechanical abilities, robust weather resistance, efficient thermal conductivity, lower density, and superior damping properties [[14], [15], [16]].

Composite foams made of polyurethane and inorganic nanoparticles are a novel class of materials in which the inorganic nanoparticles play not only the role of mechanical enhancer, but also contribute to the improvement of thermal, acoustic, and electric properties of PUR [14]. Relatively recent studies involved the use of magnetite Fe_3_O_4_ nanoparticles [17], Ag nanoparticles [18], TiO_2_ nanoparticles [19], SiO_2_ nanoparticles [20], or Al_2_O_3_ nanoparticles [21] in which the size and shape of the reinforcing agent influence a series of properties of PUR.

Considering that the main purpose in the last decades is to develop new innovative products with added value, the objective of our work consists in the valorization of PET degraded products on one hand, and use of various inorganic, micro- or nanometric wastes as filling agents to design reinforced PUR foams with special mechanical and thermal properties on the other. For this reason, we selected the most abundant by-products resulting from solid fuel and glass processing industries: fly ash and glass waste. The resulting hybrid products will have a good impact in terms of the environmental processing of waste, ensuring at the same time an excellent quality/price ratio.

Fly ash powder is one of the main industrial wastes resulting from solid fuel (coal) burning, containing mostly Si and Al metal oxides, unburned carbon, and various inorganic compounds [22]. Fly ash is a byproduct that has been used in different applications that led to the manufacturing of added-value products. A recent study developed by *Alterary and Marei* [22] revealed that many research studies were directed to the valorization of fly ash in various industries related to soil improvement, wastewater treatment, concrete, ceramics, and zeolites production. Currently, according to Makgabutlane et al. [23] one of the biggest consumers of natural resources is represented by the construction sector, efforts being made to reuse waste materials such as ceramics, bricks or pillars (coming from demolitions or renovations) since for the production of a new ton of cement for concrete bricks the CO_2_ emissions reach 622 kg. Since fly-ash is the most abundant by-product from mining industry the greatest advantages of using this waste as filler in concrete materials not only that led to the development of concrete materials creating a new geopolymer composite class [22,24], but also gave an alternative to the disposal of a hazardous waste and manufacturing of sustainable composite products in terms of environment protection. Considering that not only in our country the price of raw-materials in the construction field almost tripled in the last three years (mostly as a consequence of COVID-19 and inflation) [25], the necessity to develop products with lower manufacturing costs it is profoundly urgent. Thus, our interest is to give various formulations that include solely fly ash and plastic wastes for the development of new products for the construction field. Our work is supported by other research studies that already proved the economic and environmental advantage of incorporating fly ash and plastics as solid wastes in concrete formulations [26,27].

According to US Environmental Protection Agency, in 2017, the United States of America abandoned almost 11.4 million tons of glass waste into landfill that needed recycling [28]. Recycling glass reduces the space in landfills that would otherwise be taken up by used bottles and jars [29], while the European Container Glass Federation demonstrated that when 10 % more cullet was added into a furnace, the CO_2_ emissions were reduced by 5 % compared with the glass manufacturing process that uses only raw materials. Making glass from recycled glass will therefore, in terms of the environment, cut water pollution up to 50 % and air pollution by 20 % [30]. The European recycling strategy in the case of glass led to the production of different glass-based materials that already contain 52 % of recycled glass, while other research studies successfully utilized glass or glass wastes as binding materials in the concrete industry for geopolymer synthesis [30,31].

A recent study [32] uses fly ash from fluidized bed combustion - a waste product that is rarely utilized in industry - as a filler in modified PUR foam; the PUR foams demonstrated better thermal stability, lower carbon levels, and improved calorific values with up to 20 wt percent of filler. Thus, our study involved two steps: i) the breakdown of PET waste coming from soft-drink bottles, and ii) the development of PUR foams composites with various contents of the filling agents. The characterization of glycolysis products was performed by gel-permeation chromatography GPC analysis, while the filling agent's composition was determined by X-ray fluorescence (XRF). FT-IR testing was utilized for the characterization of the resulting hybrid PUR foams to verify the reaction among polyols and diisocyanate components. The microcellular structure was investigated by SEM, while the characteristic peaks for metal oxides from filling agents and their distribution in the PUR matrix were determined by EDX and EDX mapping. The mechanical performances were determined by compression tests, respectively the thermal behavior by TGA and DSC.

Compared to previous literature data, the originality of our research is based on the utilization of glycolysis products resulting from the depolymerization of PET and fly ash, respectively glass, all industrial wastes. In our country, PET is mostly recycled by quaternary recycling strategy (by burning the waste to produce energy) and rarely converted into commercial added value products this study giving an alternative for the valorization of plastic waste, fly ash and glass waste for development of flexible PUR foams with possible applications in automotive and civil engineering.

## 2 Materials and methods

### 2.1 Materials

Polyethylene terephthalate (PET) with a molecular weight of Mn = 25 000 g/mol was utilized in the form of flakes from post-consumer bottles. Diethylene glycol (DEG) (Fluka), zinc acetate (Fluka), methylene diphenyl diisocyanate (MDI) (Aldrich), and castor oil (Aldrich) were used as received. For this study, a local coal-fired thermal power station supplied the fly ash (Bucharest-Romania) that was used as such. The waste glass was provided by a local (Bucharest-Romania) electrical and electronic equipment waste processing facility and consisted of glass cullet up to 1 cm in size derived from TV sets and monitors out of use equipped with cathode ray tubes (CRTs). Prior to use, the glass cullet was crushed until the particles were smaller than 75 μm. In Table 1, the oxide composition of both fillers is given.

**Table 1:** The elemental composition of filling agents.

XRF analysis	Fly ash	Waste glass
Oxides	wt. %	
**SiO**_**2**_	47.4	47.4
**Al**_**2**_**O**_**3**_	24.8	5.70
**CaO**	3.30	1.50
**Fe**_**2**_**O**_**3**_	11	0.15
**SO**_**3**_	2.20	7.60
**MgO**	2.30	0.0
**K**_**2**_**O**	2.42	6.15
**TiO**_**2**_	0.87	0.00
**Na**_**2**_**O**	4.00	5.00
**P**_**2**_**O**_**5**_	0.00	0.2
**SrO**	0.00	2.7
**MnO**	0.08	0.00
**ZnO**	0.03	0.12
**CuO**	0.02	0.04
**NiO**	0.00	0.02
**BaO**	0.00	6.97
**PbO**	0.00	12.10
**Cl**	1.60	4.50
**Br**	0.05	0.00
**Cr**_**2**_**O**_**3**_	0.04	0.00

### 2.2 Methods

#### 2.2.1 Depolymerization of PET flakes

The reaction was carried out using a thermal-resistant glass reactor equipped with an automated mechanical stirrer, condenser, time controlling device, and thermostat. The trials were based on our earlier research [33], which included the addition of 1.5 g of zinc acetate as a catalyst, 150 g of DEG, and 240 g of PET. The reaction was run for 2 h at a temperature of 220 °C with constant stirring in a nitrogen environment.

#### 2.2.2 Synthesis of PUR composite foams

PET glycolysis product, castor oil, fly ash, and crushed glass waste were combined to create Component A. After adding component B (which is composed of MDI) to component A and mixing to homogenize, the whole mixture underwent a 1-min creaming period followed by the rising step.

### 2.3 Characterization

2.3.1.GPC analysis of the glycolyzed PET.

Using gel permeation chromatography (GPC) and an Agilent Technologies PL-GPC 50 Integrated GPC/SEC System equipment fitted with a refractive index detector and tetrahydrofuran (THF) as solvent, the molecular weight of depolymerized PET was determined.

#### 2.3.1 Rheometric measurements of depolymerized PET and component A

The fluid flow characteristics were obtained at 25 °C using a coaxial cylinder rheometer Rheotest 2 equipped with rotational cylinder “H” specific for fluids with high viscosities for the depolymerized PET and Component A. Shear stress (Pa) was plotted as a function of shear rate (1/s) and the rheological parameters, consistence index (a) and flow index (n) were determined based on Ostwald-de-Waele model or using the power law fluid Equation (1):

(1) $$\tau =a∙{\left(\frac{\partial u}{\partial y}\right)}^{n}$$

in which **τ** is the shear stress (Pa), **a** is the flow consistency index (Pa·s^n^), $\frac{\partial u}{\partial y}$ is the shear rate (also referred as $\dot{\gamma }$), and **n** is the dimensionless flow index. To determine the shear thinning properties of the depolymerized PET, the apparent viscosity was determined based on Equation (2):

(2) $$\mu_{app}=a∙{\left(\frac{\partial u}{\partial y}\right)}^{n-1}$$

where $\mu_{app}$ is the apparent viscosity as a function of shear rate.

According to the literature data the viscosity values were recorded after a predetermined amount of time (typically 30 s) that was required for the measurement to stabilize [34,35]. The measured values had a 1 % variation.

#### 2.3.2 X-ray fluorescence (XRF) characterization of the glass waste and fly ash

The XRF analysis, used to determine the oxide composition of both fly ash and waste glass waste, was performed by Philips PW 4025 MiniPal spectrophotometer. In Table 1 the elements found in each filling agent are presented. The ratio of SiO_2_/Al_2_O_3_ weight in fly ash is 1.91, while for glass waste is 8.51. Based on the XRF analysis, the fly ash is class F included considering that the weight ratio of SiO_2_, Al_2_O_3_, and Fe_2_O_3_ is above 70 % proving that the supplied fly ash is pozzolanic coming from bituminous or anthracite burning of coal [22].

#### 2.3.3 FT-IR analysis of polyurethane composite foams

Using a Spectrum Two FT-IR spectrometer (PerkinElmer) with a MIRacleTM Single Reflection ATR-PIKE Technologies, the FT-IR analysis was carried out at 4 cm^−1^ resolution, summing 16 scans in the 4000-550 cm^−1^ region.

#### 2.3.4 SEM and EDX analysis of the polyurethane composite foams

The morphological and structural characteristics of polyurethane foams were obtained using an element energy dispersive spectroscopy (EDS) system (Smart Insight AMETEK) at an acceleration voltage of 15 kV and a Nova NanoSEM 630 Scanning Electron Microscope (FEI Company, Hillsboro, OR, USA) at an acceleration voltage of 10 kV. By measuring about 100 individual pores, the pore distribution of polyurethane specimens loaded with fly ash and glass waste as well as blank polyurethane foams was retrieved from SEM pictures. The histograms showed a unimodal distribution of the pores and were best matched with the Gauss function.

#### 2.3.5 Density measurements for blank PUR and modified PUR composite foams

The Standard Test Method for Determination of Volume and Density of Rigid and Irregularity Shaped Molded Cellular Materials (ASTM D7710-14) was used to calculate the densities of the blank PUR and PUR foams samples treated with fly ash and glass waste. The average values were documented in Table S2 from the Supplementary file after the measurements were performed in triplicate.

#### 2.3.6 Compressive testing of the polyurethane composite foams

An Instron 3382 instrument with a 100 kN cell was used to perform mechanical compressive testing on cylindrical samples at a compression rate of 2 mm/min at room temperature. The sample's approximate measurements were 6 mm in height and 19 mm in diameter.

#### 2.3.7 Thermogravometric (TGA/DTG) analysis of the polyurethane composite foams

Thermogravimetric analysis (TGA/DTG) was carried out using NETZSCH TG 209F1 Libra apparatus. Samples weighing approximately 5 mg were heated at a rate of 10 °C/min while nitrogen flow was present.

#### 2.3.8 DSC analysis of the polyurethane composite foams

On a NETZSCH DSC 204 F1 Phoenix device, differential scanning calorimetry (DSC) investigations were carried out. 10 mg samples were heated twice, once at −50 ± 250 °C for the first heat and once at −50 ÷ 350 °C for the second heat, using nitrogen flow at a rate of 10 °C/min. Tg was calculated using the second heating.

## 3 Results and discussion

### 3.1 PET-based glycolysis product characterization and synthesis of polyurethane composite foams

The initial step of this research included the depolymerization of PET flakes and the characterization of the glycolysis products. The kinetics of the reaction was presented by our group elsewhere [6]. Based on the previous experience, the PET flakes coming from soft-drinks bottles were degraded in DEG as a glycolysis agent, and using zinc acetate as catalyst at a temperature of 220 °C for 2 h under a nitrogen atmosphere (as detailed in Section 2.2.1). The glycolysis products were investigated by GPC analysis to determine the molecular weight (Mn) and the dispersity index (PD) of the final product. Thus, the PET glycolysis product reached a molecular weight of 420 g/mol, while the PD was 1.2. These results are considered important for the next step of our study which consisted in the formulation of PURs foams built on the Mn values registered by polyol resulting from the depolymerization of PET. Thus, the synthesis of the PUR foams involved the use of two industrial wastes (fly ash, and glass) as filling agents while keeping a constant molar ratio of 1.3 of the NCO/OH groups as presented in Table 2. The content of the two filling agents varied from 4 up to 8 %, the codes representing the concentration of the filler.

**Table 2:** The composition of the samples.

Sample codes	Polyol from PET, g	MDI, g	Castor oil, g	Fly ash, g	Glass waste, g
**Blank sample**	2	2	1	–	–
**PUR-fa-4**	2	2	1	0.2	–
**PUR-fa-6**	2	2	1	0.3	–
**PUR-fa-8**	2	2	1	0.4	–
**PUR-gw-4**	2	2	1	–	0.2
**PUR-gw-6**	2	2	1	–	0.3
**PUR-gw-8**	2	2	1	–	0.4

### 3.2 Rheological results of depolymerized PET and component A

The rheological properties of fluids are crucial for the raw materials when manufacturing PUR foams at the industrial level since most industrial fluids are non-Newtonian, meaning that their viscosity will vary as a function of shear rate or shear stress. Thus, in this study we characterized the rheological behavior of depolymerized PET, of PET mixed with castor oil, and Component A.

According to literature data PET glycolyzed products have non-Newtonian behavior and are pseudoplastic [36]. In order to characterize our depolymerized PET (assigned as POL), rheological tests were recorded at 25 °C registering the shear stress (Pa) as a function of shear rate (1/s).

The rheological measurements performed on the glycolyzed PET flakes indicated that shear stress (Pa) increased as the shear rate was increased (Fig. S1 - *Supplementary file*).

According with the data mentioned in Figs. S1–a, parameter **n** (determined by applying Equation (1) as described in Section 2.3.2) has a value lower than 1 which denotes that the POL product has a pseudoplastic behavior. For POL product, the apparent viscosity, **μ**_**app**_, was calculated using Equation (2), and the apparent viscosity vs. shear rate graph (Figs. S1–b) showed a shear-thinning trend as the shear rate increased and the viscosity decreased.

Determining the shear stress as a function of shear rate was the next step for the depolymerized PET mixed with castor oil since this part is important for the blank PUR foams (formulated without filling agents).

Figs. S2–a indicated that the addition of castor oil greatly influenced the behavior of POL changing completely the flowing characteristic from a pseudoplastic shear-thinning fluid to a Bingham fluid this being attributed probably to hydrogen or electrostatic bonds between the two components [37]. Thus, the POL-castor oil blend behaves as an elastic solid under low stresses and flows as a viscous liquid at higher stress. Data from the literature indicates that there is a linear correlation between shear stress and shear rate after the fluid begins to flow [38] which was also confirmed in our case (Figs. S2–a).

Equation (3), which is also shown in Figs. S2–a, predicts that the fluid will flow at a critical shear rate (in this case, parameter a) [39,40]. Parameter **y** is the shear stress, while **b** signifies the plastic viscosity, and **x** denotes the shear rate.

(Equation 3) y = a + b·x

Using the linear fitting curve in the stress vs. strain plot, for POL-castor oil mix, the critic flow was registered at a value of almost 109 Pa.

Similar characteristics were obtained when the filling agents were added. Higher viscosities are often determined by the addition of fillers; this rise occasionally occurs over 40 % vol. concentration of the filling agent [41]. However, in our case, regardless of the filling agent or its concentration the Bingham plastic behavior of Component A was analogous with the POL-castor oil mixture (Figure S2-b, c, d, e, f, and **g**). Consequently, the concentration of the filling agent in our formulations did not influence the rheological behavior keeping the Bingham-like characteristic of Component A for all specimens. The values at which Component A starts to flow varied in a quite narrow range of the shear stress (**107.25 Pa** for **PUR-fa-4** to **109.9 Pa** for **PUR-fa-8**) indicating once more that the concentration of the filling agent was too low to induce any modifications in the rheological properties of POL-castor oil mix. It is worth mentioning that there was a slight increase of the critical shear stress value in series **PUR-fa-4 (107.25 Pa), PUR-fa-6 (107.8 Pa),** and **PUR-fa-8 (109.9 Pa)**, but in the case of glass waste this trend was not observed probably due to a non-uniform distribution of the filler during the stirring process of the whole compounds of Component A filled with glass waste.

### 3.3 FT-IR analysis of polyurethane composite foams

**Fig. 1:**
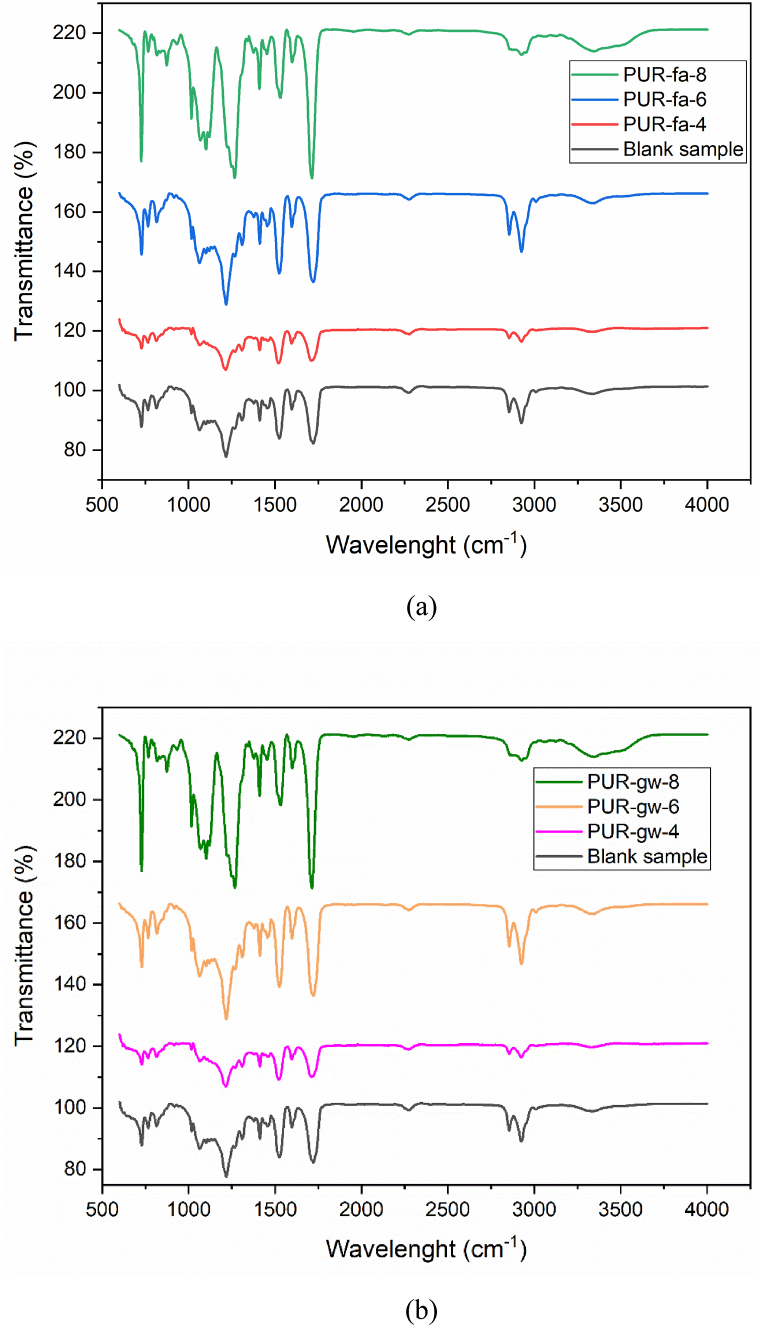
FT-IR results of the blank PUR sample (black line), fly ash-based (a), and glass waste-based PUR foams (b).

The polyurethane samples were examined using FT-IR analysis to demonstrate the reactivity between the isocyanate component and the PET-based polyol as depicted in Fig. 1.

In Fig. 1a and b the FT-IR analysis evidenced the characteristic vibrations of polyurethane structure for all samples filled with fly ash [32] and glass waste. The signal from 3332 cm^−1^ was assigned to NH stretching and the peak from 1721 cm^−1^ attributed to C=O stretching confirmed the formation of urethane groups due to the reaction between the OH groups and NCO groups from MDI [32]. Vibrations of CH_2_, respectively CH_3_ groups were registered at 2925 cm^−1^ and 2857 cm^−1^. The terminal groups of the polymeric chain were identified as the source of the NCO group seen at 2267 cm^−1^. The aromatic rings of the MDI hard segments or those from the terephthalate unit can be attributed to the C=C recorded at 1598 cm^−1^. Furthermore, the multiple bonds in castor oil's fatty acid-based composition can also be responsible for this signal. The N–H bending in the amide group's plane was identified as the reason for the peak at 1520 cm^−1^. The CN stretching vibrations in amine designated at 1219 cm^−1^provided another proof that the process occurred [42].

To evidence the presence of the fillers, an amplification of the possible characteristic signals was performed by FT-IR analysis. In Fig. S3 (Supplementary file) the characteristic signals of Si–O group (1018 cm^−1^), Si–OH group (872 cm^−1^), and silica bond (820 cm^−1^), were identified for fly ash [43]. In the case of glass waste, Fig. S4 (Supplementary file), by FT-IR analysis the signal of Si–*O*–Si was registered at 1271 cm^−1^, the peak for Si–O at 1018 cm^−1^, and silica bond at 817 cm-1 [43]. These outcomes and the XRF analysis agree well confirming the presence of SiO_2_ in both filling agents (Section 2.3.2 – Table 1).

### 3.4 SEM and EDX analysis of the polyurethane composite foams

**Fig. 2:**
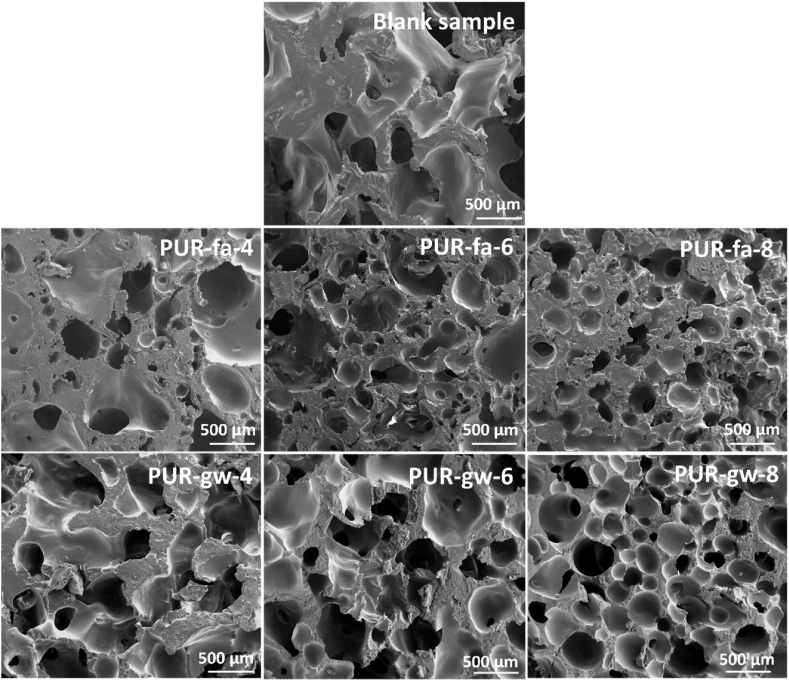
SEM micrographs of the blank polyurethane foam (blank sample) and polyurethane specimens filled with fly ash (Samples PUR-fa-4, PUR-fa-6, and PUR-fa-8) respectively waste glass (Samples PUR-gw-4, PUR-gw-6, and PUR-gw-8).

In Fig. 2, the SEM micrographs indicate a close cellular structure specific to polyurethane foams [44]. However, as the filling agent concentration increased, the pores dimension decreased. In the case of polyurethane foams filled with fly ash, the images from Fig. 2 – b, c, d performed at the same magnification revealed that the pores of **PUR-fa-8** are smaller compared to the blank sample and the other two counterparts (**PUR-fa-4** and **PUR-fa-6**). The same behavior was noticed for the specimen with glass waste as a filling agent (Fig. 2 – e, f, g) in which the smallest pores were formed at a higher concentration of the filling agent (**PUR-qw-8**), compared with **PUR-gw-4** and **PUR-gw-6**. Furthermore, when the filling agent concentration was raised, not only were the pores smaller than in the blank sample, but this parameter also resulted in an increase in the total number of pores. Thus, **PUR-fa-8**, respectively **PUR-qw-8** presented the highest number of pores in the SEM images (Fig. 2-d, respectively Fig. 2-g).

In the case of PUR foams composites formulated with the two types of filling agents, the mechanical performances of the materials could be substantially influenced by the filling agent's distribution in the whole polymer matrix. For this reason, EDX mapping analysis was performed for all samples to investigate the spreading of fly ash or glass waste in the polyurethane composition. EDX mapping revealed the distribution of the most intense signals attributed to different metal oxides identified in the chemical composition of the two types of wastes by XRF analysis (section 3.1).

All samples' EDX spectra are displayed, with the number of counts on the y-axis and energy in keV on the x-axis. In Energy Dispersive Spectroscopy (EDS), the "K line" refers to the characteristic X-ray emissions that occur when an inner-shell electron vacancy in an atom is filled by an electron from a higher energy level.

**Fig. 3:**
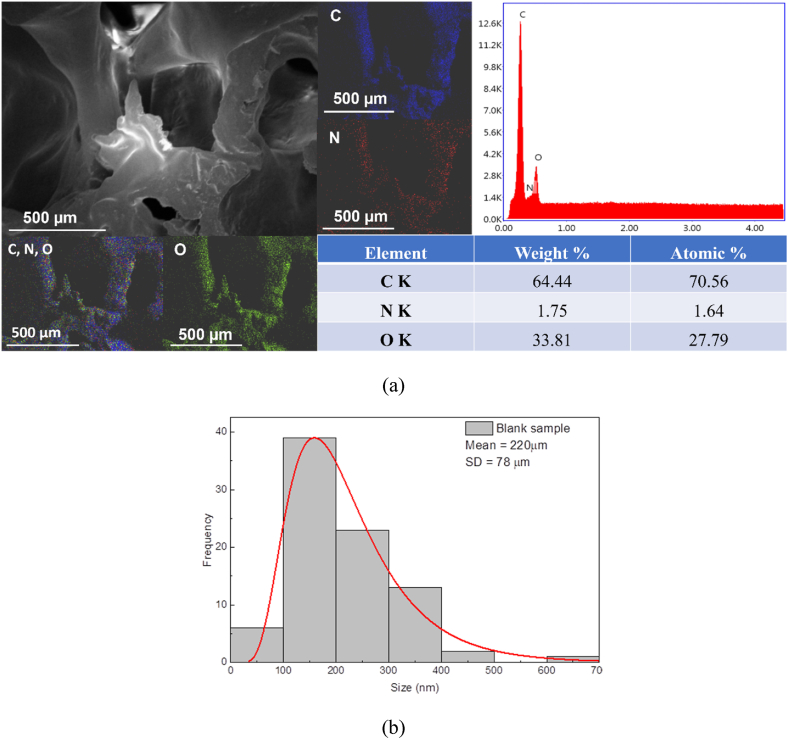
EDX spectrum and EDX mapping analysis of blank polyurethane foam.

For the Blank sample, the EDX spectrum registered, as expected, only C, N and O elements attributed strictly to the polymer matrix (Fig. 3a). According to the EDX mapping, every component in the polyurethane foam's structure was distributed uniformly. The blank sample's pore size distribution is shown in Fig. 3b. The blank polyurethane foam pore size, showed by SEM micrographs and statistical measurements, were in the range of 48 μm–635 μm. The greatest percentage of pores diameter was of 100–255 nm having the **mean diameter of 220 ± 78 nm**.

(Blank sample) (a) and pore size distribution (b)

**Fig. 4:**
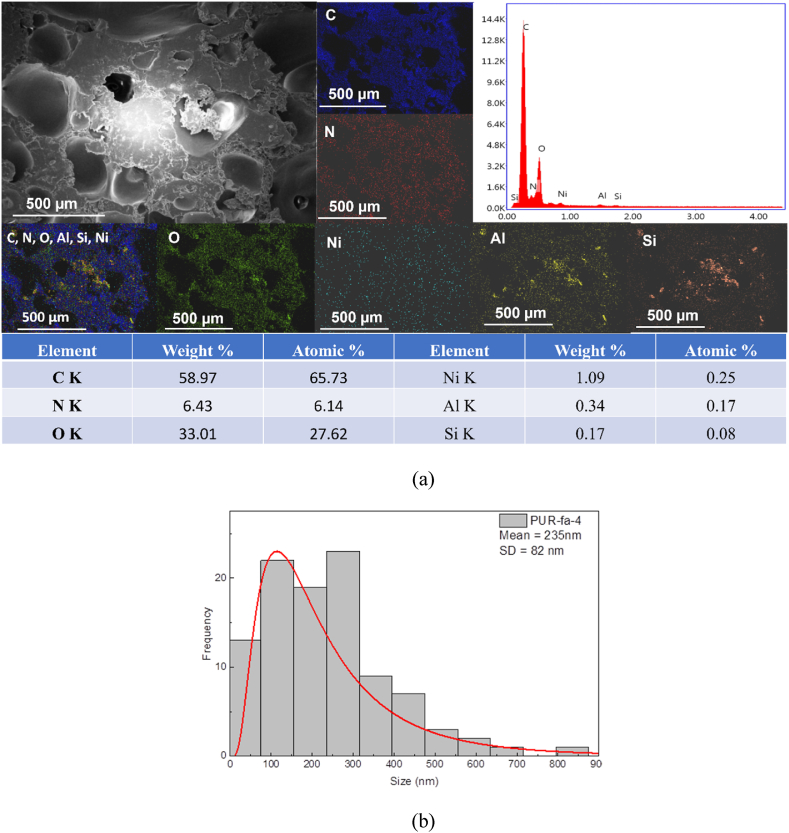
EDX spectrum and EDX mapping analysis of polyurethane foam.

Samples **PUR-fa-4**, **PUR-fa-6**, and **PUR-fa-8**, obtained by adding different amounts of fly ash registered strong intense peaks for C, N and O attributed to the polyurethane matrix as confirmed by all spectra from Fig. 4, respectively Figs. S5 and S6 (Supplementary file), respectively. The additional signals that were registered were attributed to Ni, Al and Si. As presented, the weight of the elements varies according to the preparation of the PUR foam. Thus, in the case of **PUR-fa-4**, C weight % was lower compared to the blank sample, while the new peaks of Ni, Al and Si from NiO, Al_2_O_3_ and SiO_2_ oxides confirmed the presence of the fly ash filling agent. However, it is worth mentioning that in the case of fly ash the Ni was not detectable, while in the case of EDX spectra, Ni signal was identified in all samples. In the case of XRF analysis, this result can be explained by the lower energy lines applied for the fly ash or by the lower concentration of the elements in the samples that can decrease the level of confidence for their detection [45]. The weight percent for Al and Si varied from one sample to another registering a weight ratio of Si/Al of 0.5 for **PUR-fa-4**, 0.83 for **PUR-fa-6**, respectively 0.83 for **PUR-fa-8**. Based on these results we were able to notice that the ratio of the elements increased with the concentration of the filler. However, in the case of **PUR-fa-6**, respectively **PUR-fa-8**, the Si/Al weight ratio was identical. This can be attributed to the different distribution of the elements from the fly ash into the polymer matrix considering that relatively small parts of the surface samples are exposed to EDX analysis. This aspect is confirmed by the EDX mapping as well since all the elements are distributed differently and agglomeration/higher concentration of fly ash was noticed in several zones/areas of the sample surface for Si and Al. Nevertheless, the other elements have a relatively uniform distribution on the surface regardless of the increasing concentration of fly ash (Fig. 4a and b, S5, and S6 – EDX mapping) with some areas in which Al signal is more intense compared to Ni, and Si elements probably due to some agglomerations of the fly ash during mixing or rising step of the polyurethane formulation.

filled with fly ash (a) and pore size distribution (b) for **PUR-fa-4**.

The pore size of the polyurethane specimens filled with fly ash Samples **PUR-fa-4, PUR-fa-6, and PUR-fa-8** (Fig. 4, respectively S7 and S8) was registered based on SEM micrographs and statistical data, ranging from 23 μm to 800 μm, 28 μm–750 μm, and 30 μm–444 μm, respectively. The highest percentage of pores were found to be within 98–302 μm, 46–198 μm, 51–202 μm diameter pore range having the **mean diameter 235 ± 82** μm, **178 ± 65** μm, **178 ± 58** μm, respectively.

**Fig. 5:**
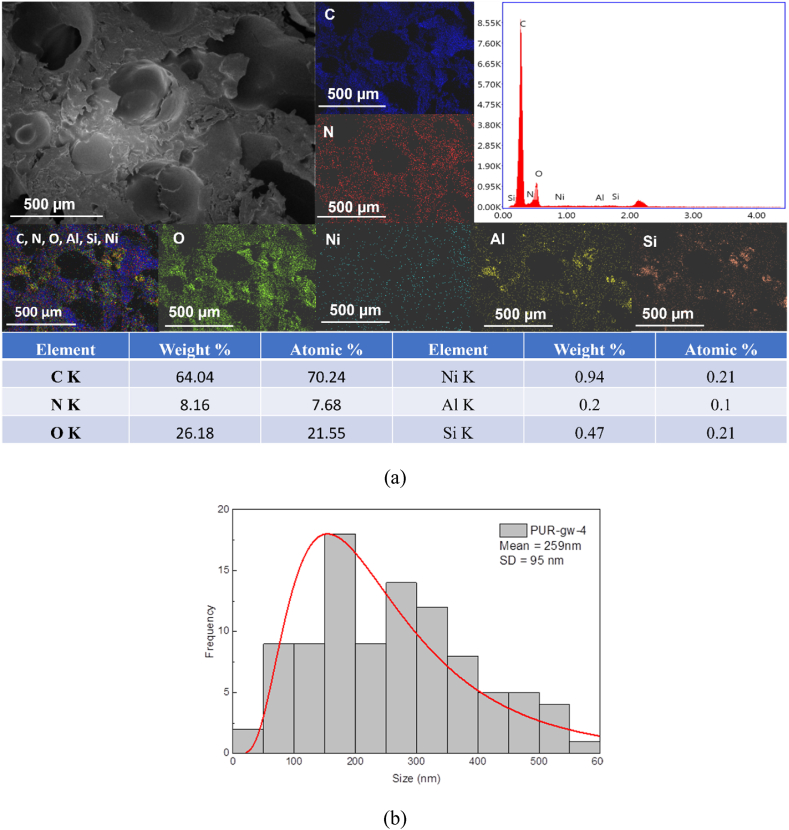
EDX spectrum and EDX mapping analysis of polyurethane foam.

In the case of using glass waste as filler, Samples **PUR-gw-4**, **PUR-gw-6**, and **PUR-gw-8** had similar behavior. The weight ratio of Si/Al was 2.33 for **PUR-gw-4**, 0.23 for **PUR-gw-6**, and **PUR-fa-8** for **PUR-gw-8** (Fig. 5a and b, S7, and S8). Thus, in this case, the weight ratio of Si/Al increased as the concentration of filler was increased in **PUR-gw-4** and **PUR-gw-8**. However, in **PUR-gw-6**, the lower ratio between elements can be attributed to the exposal of a limited part of the sample (Fig. S7) and the increase of the filling agent concentration did not increase the weight ratio of Si/Al compared with the other two samples filled with glass waste.

By EDX mapping, for **PUR-gw-4** it was noticed that Al and Si elements are more agglomerated in a different zone of the samples surface (Fig. 5), while for the rest of the samples, a uniform distribution of all elements was registered (Fig. S7, respectively Fig. S8). Overall, the distribution of all elements was quite uniform in all samples formulated with glass waste.

The waste glass (**PUR-gw-4, PUR-gw-6, and PUR-gw-8**) pore size distribution was determined by the SEM analysis, ranging from 26 μm to 561 μm, 27 μm–754 μm, and 49 μm–523 μm, respectively. The highest percentage of pores were found to be within 89–269 μm, 51–225 μm, 63–201 μm diameter pore range having the **mean diameter 259 ± 95** μm, **233 ± 105** μm, **182 ± 75** μm, respectively.

filled with glass waste (a) and pore size distribution (b) for **PUR-gw-4**.

**Fig. 6:**
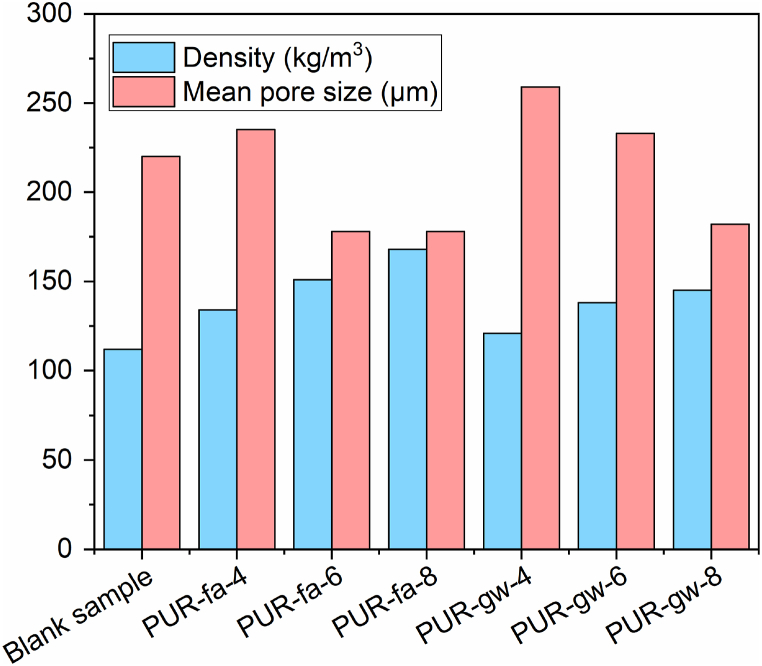
The mean values for pore size and density for all PUR specimens.

In Table S1 are presented the data obtained for the pore size distribution for all specimens in a concise manner to highlight the difference between the samples. To give a correlation between the mean values of pore size and density were plotted for all specimens as shown in Fig. 6. The values for density (table S2 – Supplementary file) in the case of the specimens modified with fly ash increased as the content of the filler was increased from sample **PUR-fa-4** to **PUR-fa-8**, while the pores size mean values decreased. The same trend was observed also in the case of the PUR foams modified with glass waste. The values of the density in the case of glass waste-based samples were lower compared with those filled with fly ash, this being attributed to the filler's particle size (Fig. S9). It is worth mentioning that this correlation is in good agreement with literature data [46] and the values obtained for density ranging from 112 kg/m^3^ (Blank sample) to 168 kg/m^3^ (PUR-fa-8) are specific for flexible foams [47]. **In** Table S3 **(Supplementary file) are presented the SEM micrographs performed at 100X in different areas of each sample in order to put into evidence the homogeneity of the samples. Thus, it can be noticed that the samples are not homogeneous.**

### 3.5 Compressive testing of the polyurethane composite foams

**Fig. 7:**
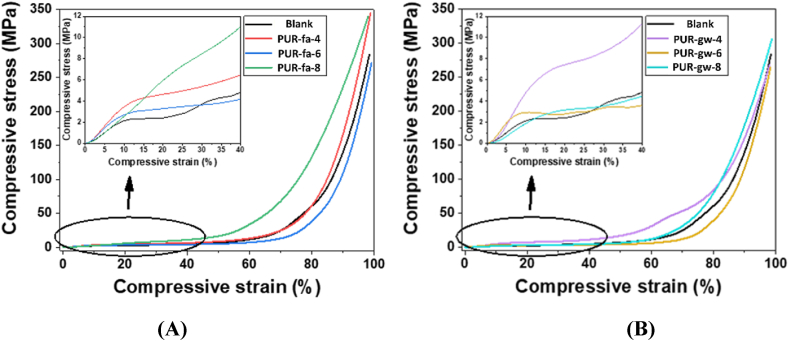
Compressive testing for blank sample and polyurethane foams filled with fly ash **(A)**, and glass waste **(B)**.

In Fig. 7, the plot of compression stress versus compression strain for the blank samples and PUR foams loaded with fly ash (Fig. 7-A) and glass waste (Fig. 7-B) was presented.

The compression mechanical tests of the PUR foams confirmed that the compression stress and compression strain increased for the specimens in which the lowest concentration of reinforcing agent was added. Thus, the best mechanical performances were registered by **PUR-fa-4** (Fig. 7-A -detailed image), respectively **PUR-gw-4** (Fig. 7-B -detailed image). The elastic, plateau, and densification areas, which were also verified by Petrů and Novák [48], are the three regions that best describe the mechanical behavior of all samples, as can be seen from the trend of the curves.

The first step of the compression test takes place from the moment the cell walls approach until they begin to collapse. In the plateau stage, a buckling of the samples under pressure occurs, while in the final step, a collapse of the cell walls happens until reaching the opposite walls, which determines the densification/compression of polyurethane foam.

The presence of fly ash and glass waste particles extended the elastic region and increased the slope of the curves due to the higher rigidity of the cellular walls. For the plateau region, the slope increased slightly due to the incorporation of the nanoparticles in the cellular structure. This led to a reinforcing of the cell walls that determined a higher mechanical resistance of the porous matrix [49]. Furthermore, the propagation of the crack seems to be delayed giving higher resistance to complete breaking/collapse. All these mechanical improvements were registered for all samples compared with the blank sample and are influenced by the lowest content of the filler and the uniform distribution of the filling agents in the structure of the PUR foams. It is worth mentioning that, as expected, by increasing the concentration of the filling agent, the mechanical properties decreased in the case of Samples **PUR-fa-6**, **PUR-fa-8**, **PUR-gw-6** and **PUR-gw-8**. The high value registered for the compressive stress (200–300 MPa), indicates that these types of materials may be suitable for use in the civil engineering industry [[50], [51], [52]].

**Fig. 8:**
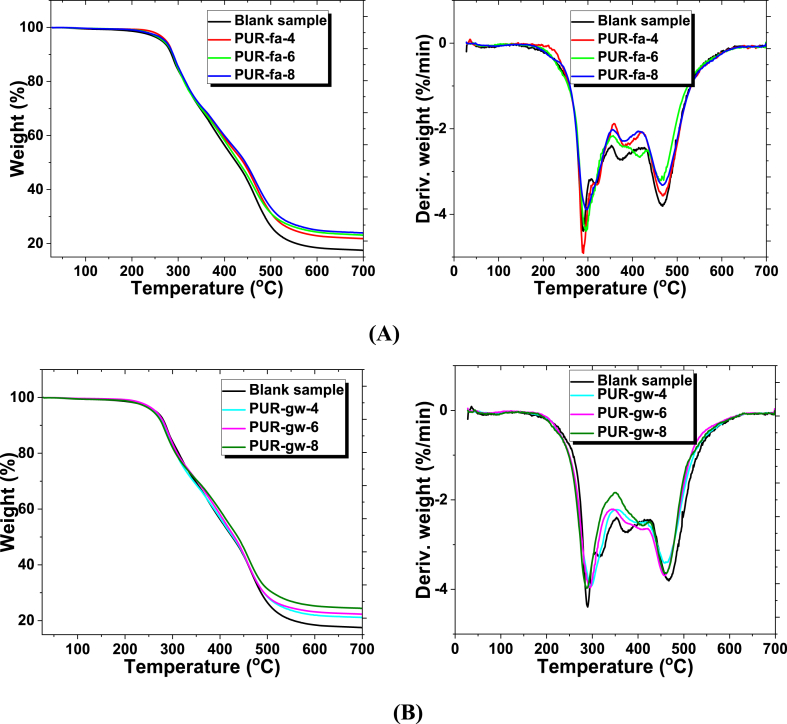
TGA and DTA analysis of blank sample and polyurethane foams loaded with fly ash **(A)**, and glass waste **(B)**.

To sustain the possible applications of formulated PURs, supplementary information can be obtained by TGA and DSC analysis. In Fig. 8A and **B** two decomposition stages were identified in the range of 230–320 °C, respectively 450–480 °C and attributed to the characteristic two step decomposition process of urethane bonds [53]. Between 320 and 400 °C the Gram-Schmidt trend was identified for the degradation of volatile compounds [54]. After 480 °C, the polymer matrix was completely degraded. The mass residue increased with the filling agent concentration. Based on the filling agent's concentration we did not register any other differences except the residual mass. Since the filling agent and polymer chains only interact physically and not chemically, the outcomes are predictable.

**Fig. 9:**
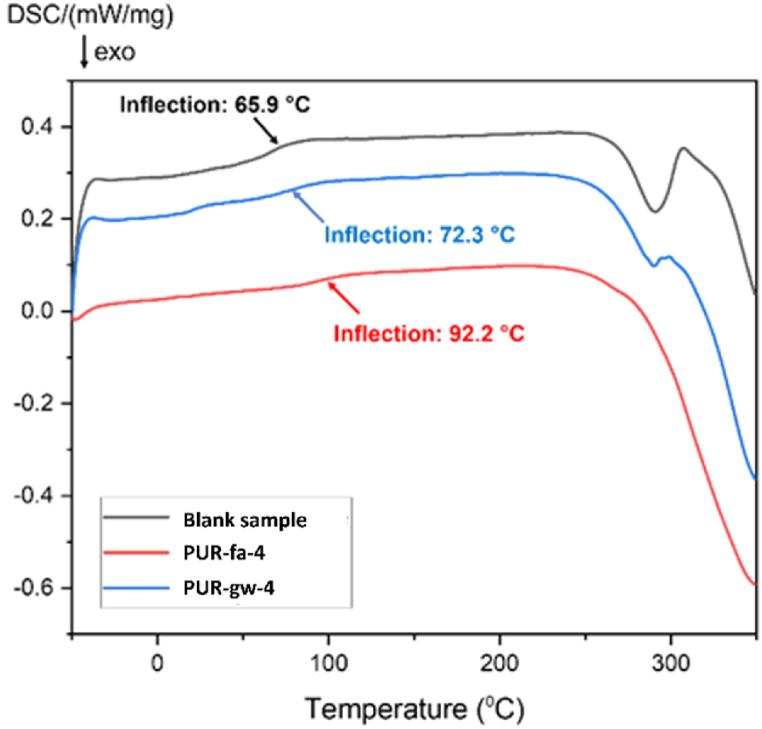
DSC diagram for the Blank sample, **PUR-fa-4**, and **PUR-gw-4**.

By DSC analysis (Fig. 9), glass transition temperature (Tg) was determined for all samples that registered the best results in terms of mechanical performance (**PUR-fa-4**, respectively **PUR-gw-4**) comparatively with Blank sample.

For **PUR-gw-4**, the value of Tg increases compared with the value registered for Blank sample. The highest value of Tg has been registered for the composite filled with the lowest concentration of fly ash, **PUR-fa-4**.

Tg is a parameter that characterizes the movement/flexibility of the polymer chain, the value being lower with the loss of chain mobility. The increase of Tg in the case of PURs reinforced with different filling agents can be explained by preventing the mobility of polymer chains (compacting effect) that determines a more loosened structure as the temperature rises.

**Scheme 1:**
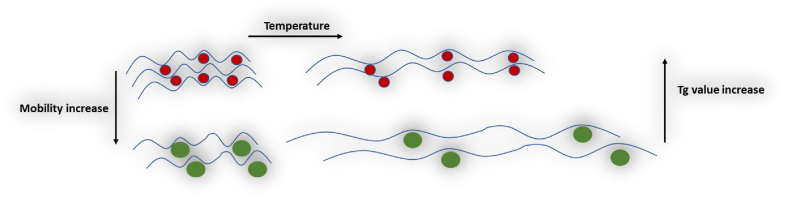
The influence of mobility to Tg value.

In Scheme 1 is presented the influence of the reinforcing agents’ dimension on the polymer chain mobility, a lower dimension of the particles (a higher specific surface) inducing a higher Tg [55]. In the case of fly ash (represented by red spheres in Scheme 1) the Tg value was 92.2 °C, while for glass waste (the green spheres) the value of Tg was lower (72.3 °C).

In the case of **PUR-fa-4**, a higher number of contact points between the polymer matrix and the reinforcing agent can occur since the fly ash has a higher specific surface area since the particles of this type of waste are spherical in majority (*Supplementary file* – Figs. S7–a), thus leading to a higher Tg value (Fig. 9) confirming the improvement of thermal properties [56].

On the contrary, for the specimen with glass waste, the irregularities and different shapes and sizes of the material (Supporting *file* – Figs. S7–b) led to a decrease of Tg (Fig. 9).

Thus, in terms of thermal characteristics, in our case the highest values of Tg were determined for the PURs formulated with the lowest concentration of fly ash.

## 4 Conclusions

This study evidenced the synthesis and characterization of flexible PUR foams manufactured from industrial wastes. Glycolysis products from PET depolymerization in the presence of DEG and castor oil represented the polyol component.

The rheologic tests revealed that degraded PET is a non-Newtonian pseudoplastic shear-thinning fluid, while the addition of castor oil changed the behavior of the mixture to a Bingham fluid. After the critical shear rate the mix acts like a Newtonian fluid. In the case of Component A resulted from the depolymerized PET, castor oil and the filling agents, the rheologic behavior was uneffected by the type of the filler or the filler's concentration maintaining the Bingham-like behavior.

FT-IR analysis evidenced the reaction between polyols and diisocyanates confirming the formation of polyurethane structure without excess of functional groups that could influence the mechanical properties of final products. SEM, EDX and EDX mapping revealed a cellular structure in which the filling agents were uniformly incorporated as confirmed by EDX mapping. In all cases, the EDX spectrum was in good accordance with XRF analysis performed for the fillers. The values of pores size of the samples decreased with the increase of the concentration of the filler. The density values ranged from 112 kg/m^3^ up to 168 kg/m^3^ and an increasing trend was observed as the concentration of the filler was increased. The values registered for density confirmed the formulation of PUR foams with flexible characteristics which were also confirmed by the compression tests regardless of the filling agent.

The compression analysis confirmed that the best mechanical performances were registered by the specimens with the lowest concentration of filling agent, while the highest thermal resistance was achieved by the PUR foams with the highest concentration of the reinforcing agent.

The TGA analysis registered two decomposition steps specific for urethane bonds and Gram-Schimdt profile for volatile compounds. The residual mass was found to be higher for the PUR foams formulated with the highest concentration of filler, irrespective of the filling material utilized.

The highest value of Tg determined by DSC analysis was obtained for the PUR specimen modified with the lowest concentration of fly ash. The value of Tg increased up to 92.2 °C for **PUR-fa-4** compared with **PUR-gw-4** (72.3 °C) being influenced by the mobility of the polymer chain assigned to the morphology and size distribution of the filling agents. The obtained flexible PUR foams in this study could be utilized in applications related with automotive industry and civil engineering.

## Data Availability statement

The authors confirm that the data supporting the findings of this study are available within the article [and/or] its supplementary materials.

## CRediT authorship contribution statement

**Adriana Cornelia Mârșolea (Cristea):** Methodology. **Alexandra Mocanu:** Formal analysis. **Paul Octavian Stănescu:** Formal analysis. **Oana Brincoveanu:** Formal analysis. **Cristina Orbeci:** Conceptualization. **Roberta Irodia:** Formal analysis. **Cristian Pîrvu:** Formal analysis. **Adrian Dinescu:** Conceptualization. **Constantin Bobirica:** Software. **Edina Rusen:** Conceptualization.

## Declaration of competing interest

The authors declare the following financial interests/personal relationships which may be considered as potential competing interests.
