# Supplementary material for: Synthesis and characterization of polyurethane flexible foams provided from PET derivatives, fly ash, and glass wastes
Source: Heliyon. 2023 Dec 1;9(12):e23097. doi: 10.1016/j.heliyon.2023.e23097 (PMC10777411; doi:10.1016/j.heliyon.2023.e23097)
Supplement: Multimedia component 1 [file mmc1.docx]

**Synthesis and characterization of polyurethane flexible foams provided from PET derivatives, fly ash, and glass wastes**

Adriana Cornelia Mârșolea (Cristea)^1^, Alexandra Mocanu^1,2^, Paul Octavian Stănescu^1,3^,

Oana Brincoveanu^2,4^, Cristina Orbeci^1^, Roberta Irodia^1^, Cristian Pîrvu^1^, Adrian Dinescu^2^, Constantin Bobirica^1^, Edina Rusen^1,*^

*^1^University Politehnica of Bucharest, Faculty of Chemical Engineering and Biotechnologies, 1- 7 Gh. Polizu Street, 011061 Bucharest, Romania*

*^2^National Institute for Research and Development in Microtechnologies – IMT Bucharest, 126A Erou Iancu Nicolae Street, 077190 Bucharest, Romania*

*^3^Advanced Polymer Materials Group, Faculty of Chemical Engineering and Biotechnology,*

*University Politehnica of Bucharest, 1-7 Gh. Polizu Street, 011061 Bucharest, Romania*

*^4^Research Institute of the University of Bucharest, ICUB Bucharest, Soseaua Panduri, nr. 90, Sector 5, 050663, Bucureşti*

**e-mail corresponding author: edina.rusen@upb.ro*

(*Supplementary information*)





(a)





(b)

**Figure S1.** Shear stress (Pa) (a) and viscosity (Pa·s) (b) as functions of shear strain (1/s) for the glycolyzed PET product





(a)





(b)





(c)





(d)





(e)





(f)





(g)

**Figure S2.** Shear stress versus shear strain and linear fit curves for the determination of rheological behavior of POL and castor oil mixture (a), Component A filled with flying ash of different concentrations (b, c, d), and Component A filled with glass waste (e, f, g)





**Figure S3.** FT-IR analysis that evidences the presence of Silica signals from fly ash





**Figure S4.** FT-IR analysis that evidences the presence of Silica signals from glass waste


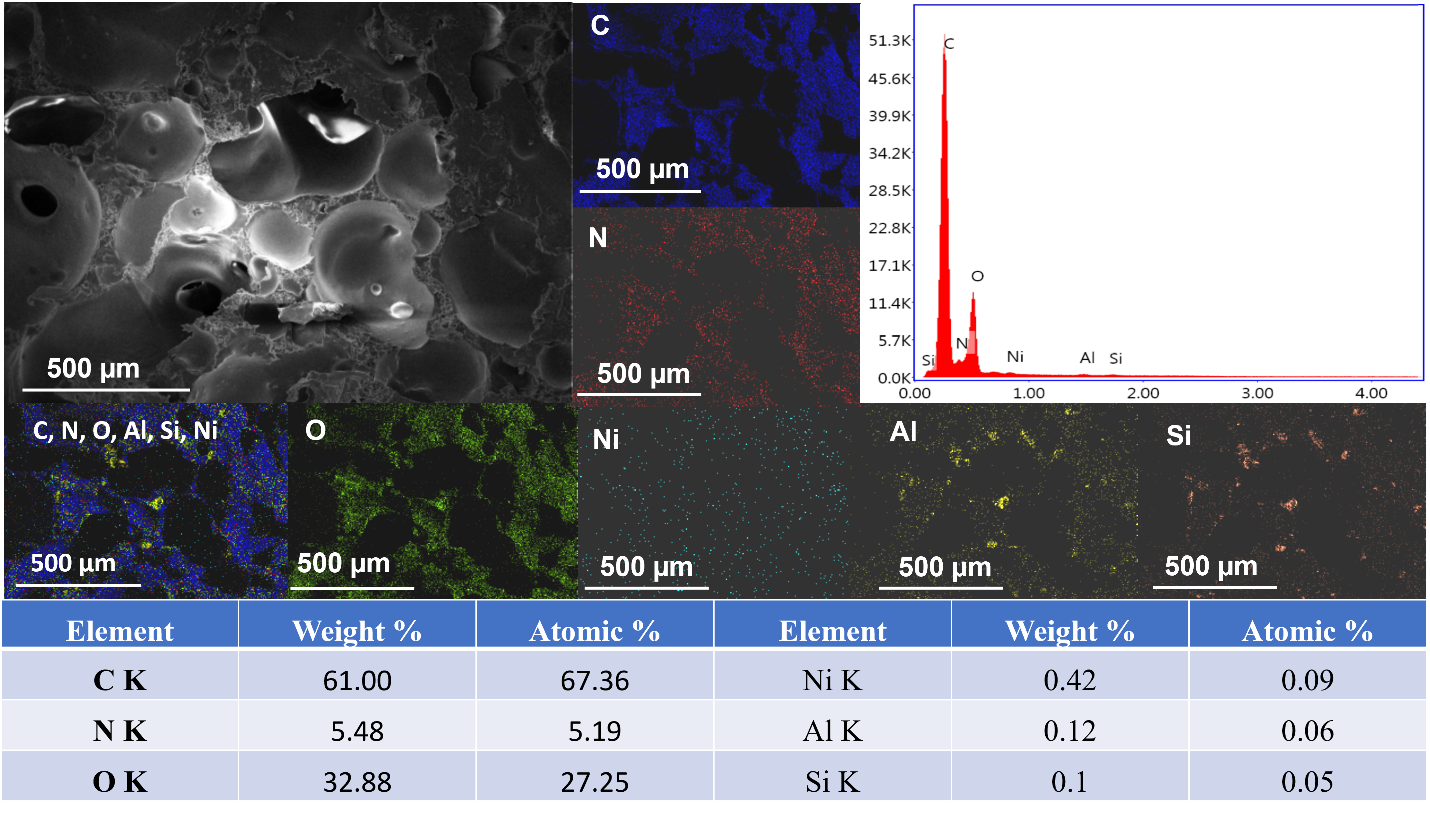


(a)


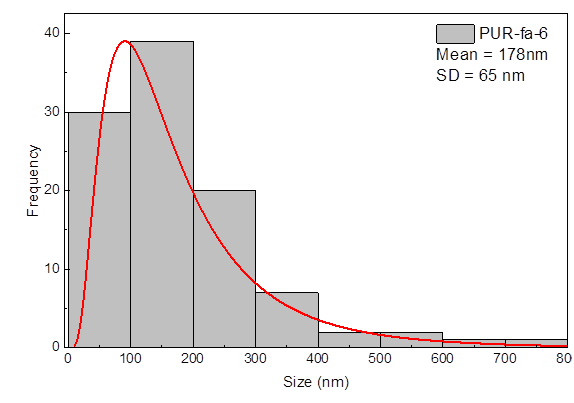


(b)

**Figure S5.** EDX spectrum and EDX mapping analysis of polyurethane foam

filled with fly ash (a) and pore size distribution (b) for **PUR-fa-6**


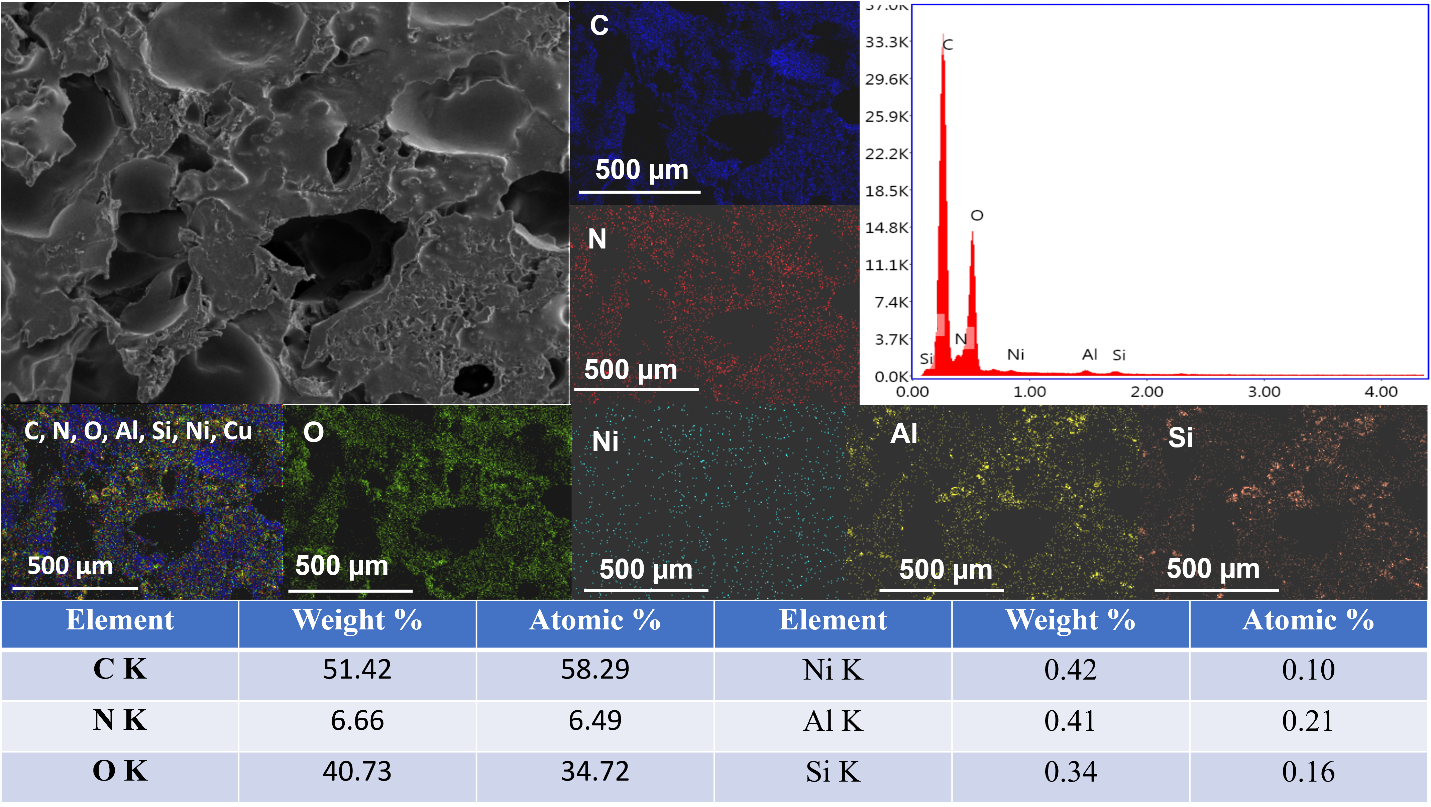


(a)


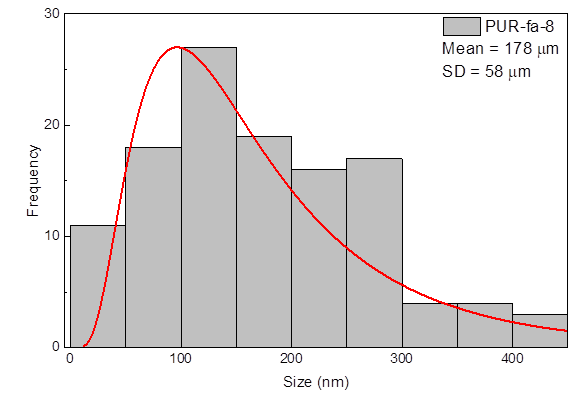


(b)

**Figure S6.** EDX spectrum and EDX mapping analysis of polyurethane foam

filled fly ash (a) and pore size distribution (b) for **PUR-fa-8**


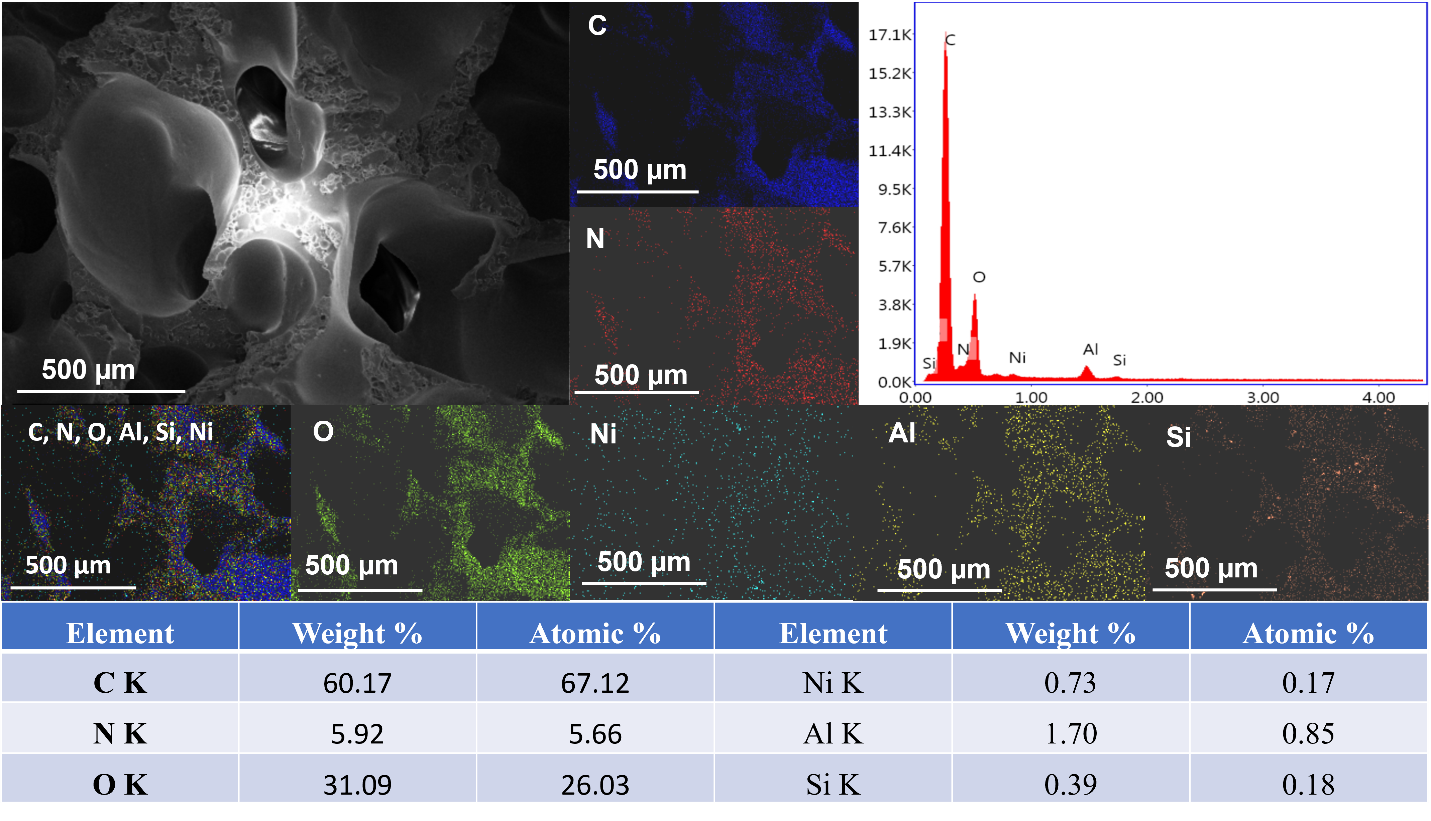


(a)


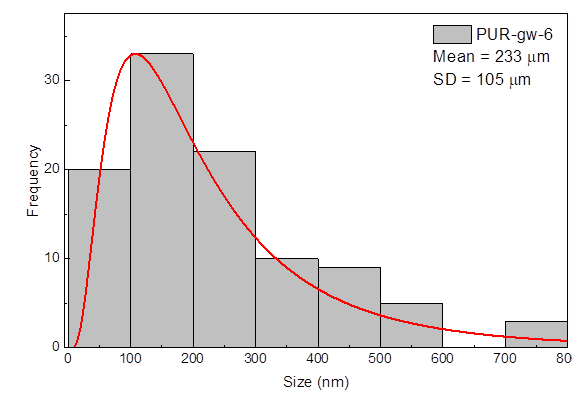


(b)

**Figure S7.** EDX spectrum and EDX mapping analysis of polyurethane foam

filled with glass waste (a) and pore size distribution (b) for **PUR-gw-6**


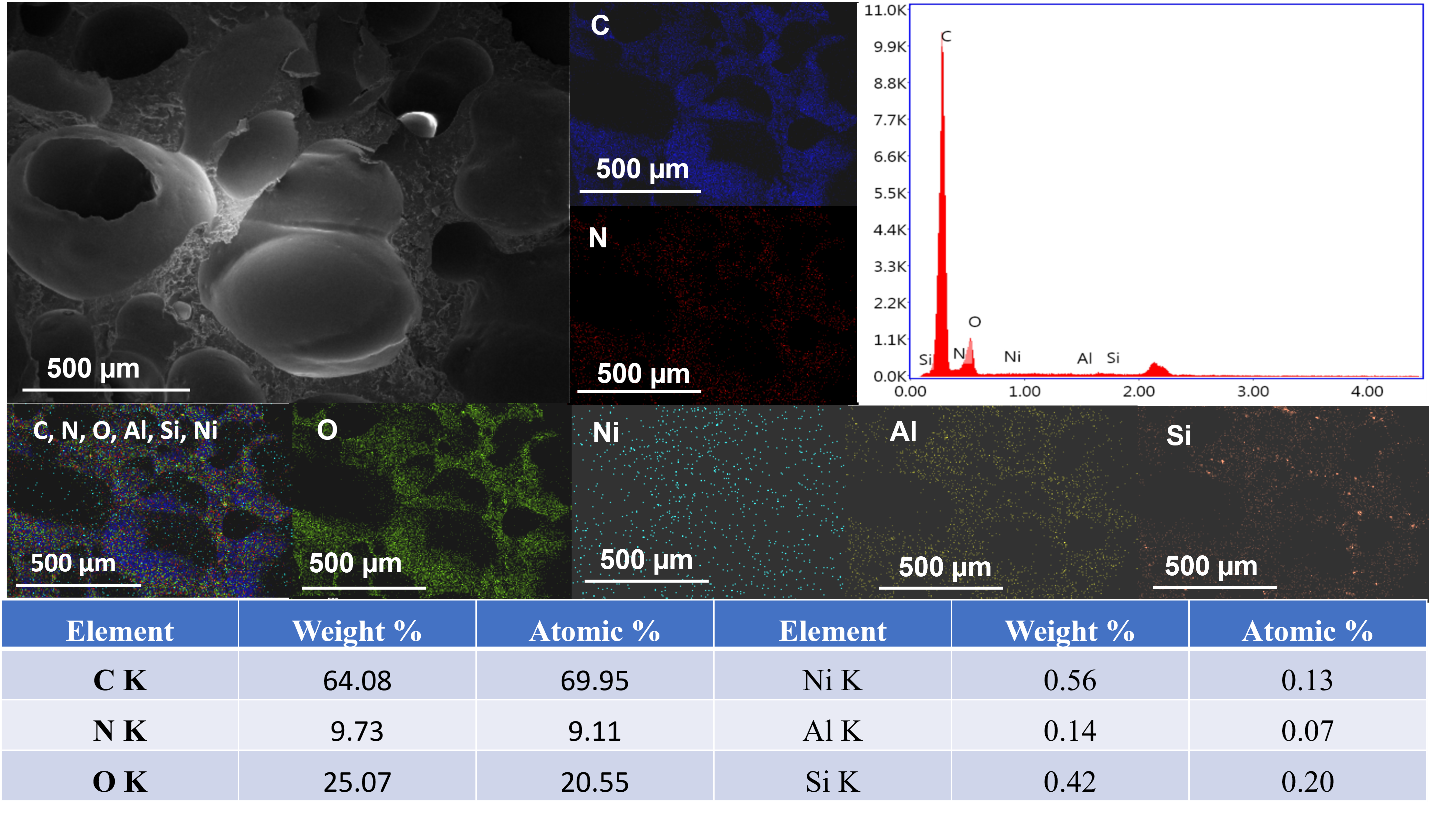


(a)


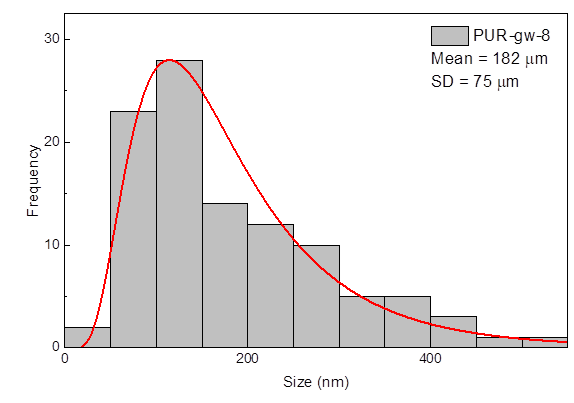


**Figure S8.** EDX spectrum and EDX mapping analysis of polyurethane foam

filled with glass waste (a) and pore size distribution (b) for **PUR-gw-8**

**Table S1**. Characterization of the PUR foams – determination of pore size distribution

| **Sample** | **Mean pore size (µm)** | **SD (µm)** | **Min-Max (µm)** | **FWHM (µm)** |
| --- | --- | --- | --- | --- |
| Blank sample | 220 | 78 | 48 -635 | 100-255 |
| **PUR-fa-4** | 235 | 82 | 23-800 | 98-302 |
| **PUR-fa-6** | 178 | 65 | 28-750 | 46-198 |
| **PUR-fa-8** | 178 | 58 | 30-444 | 51-202 |
| **PUR-gw-4** | 259 | 95 | 36-561 | 89-269 |
| **PUR-gw-6** | 233 | 105 | 27-754 | 51-225 |
| **PUR-gw-8** | 182 | 75 | 49-523 | 63-201 |

**Table S2.** *The density of the PUR specimens determined by ASTM D7710-14*

| **Sample** | **Density, kg/m^3^** | **SD, kg/m^3^** |
| --- | --- | --- |
| **Blank sample** | 112 | 4.5 |
| **PUR-fa-4** | 134 | 6.7 |
| **PUR-fa-6** | 151 | 8.3 |
| **PUR-fa-8** | 168 | 9.6 |
| **PUR-gw-4** | 121 | 4.8 |
| **PUR-gw-6** | 138 | 7.3 |
| **PUR-gw-8** | 145 | 8.1 |


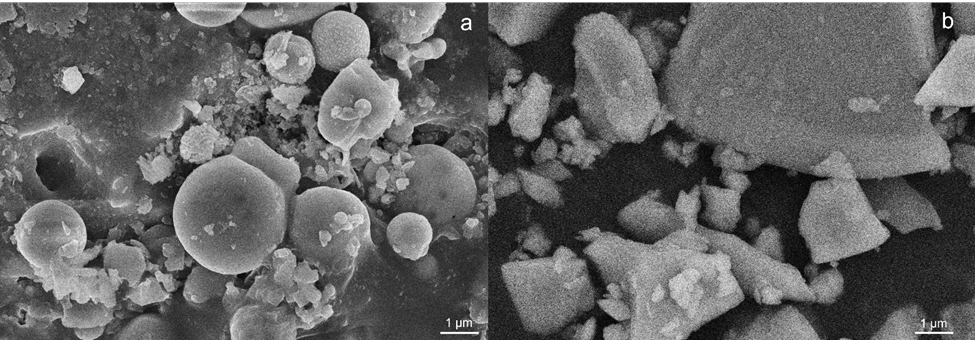


**Figure S9**. SEM micrographs of fly ash (a), and glass waste (b)

**Tabel S3.** *SEM micrographs performed at 100X in different areas of each sample*

| **BLANK SAMPLE** | | |
| --- | --- | --- |
| 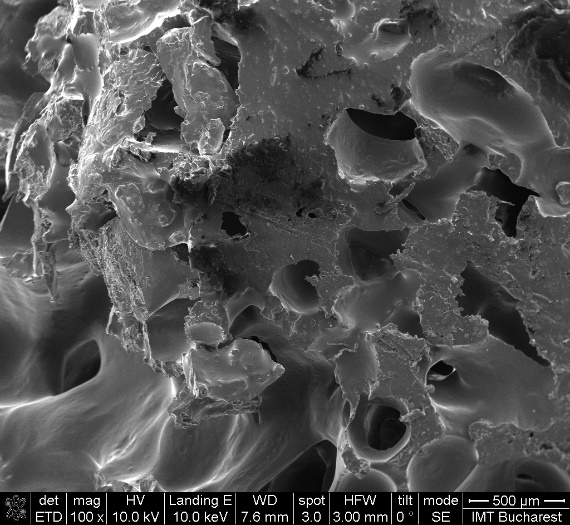 | 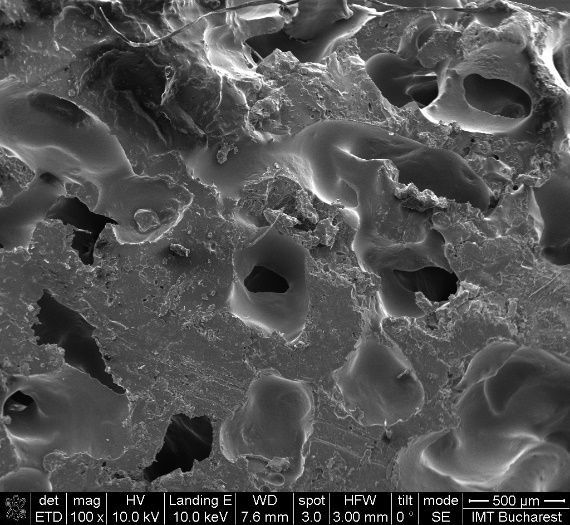 | 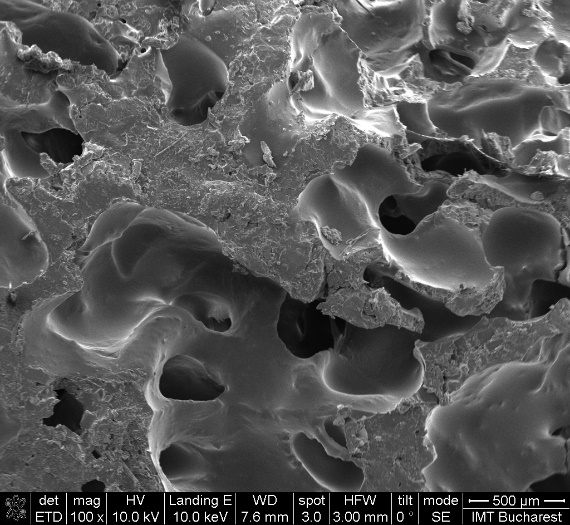 |
| 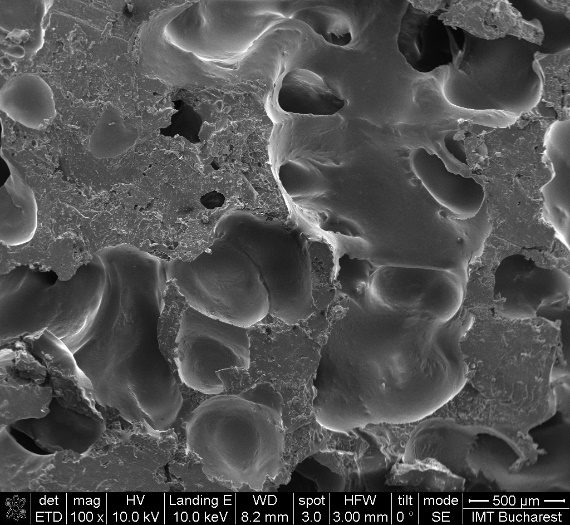 | 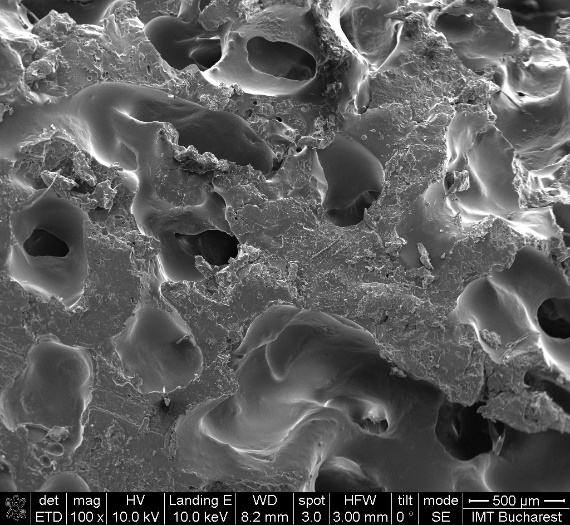 | 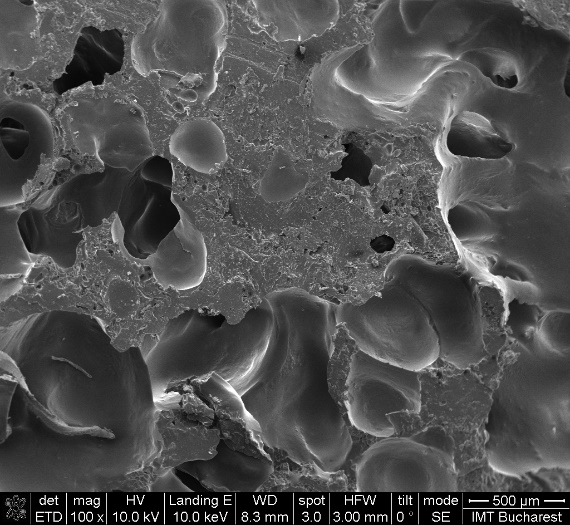 |
| **PUR-fa-4** | | |
| 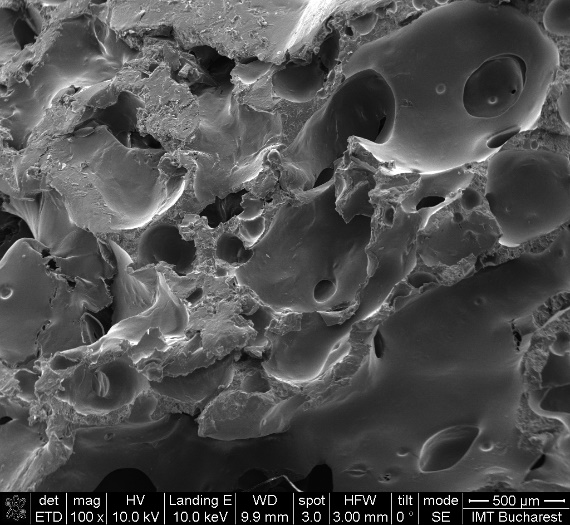 | 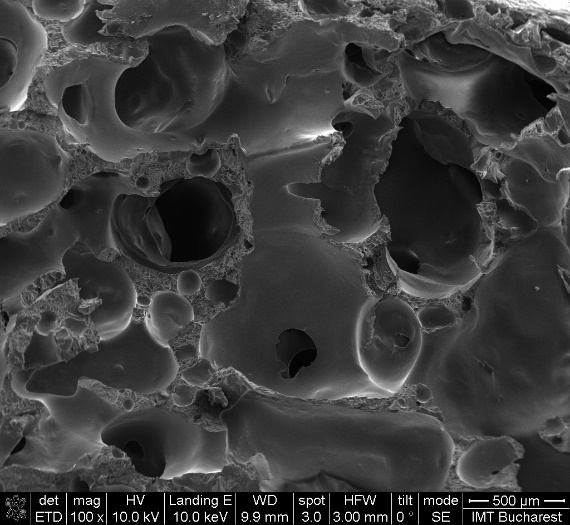 | 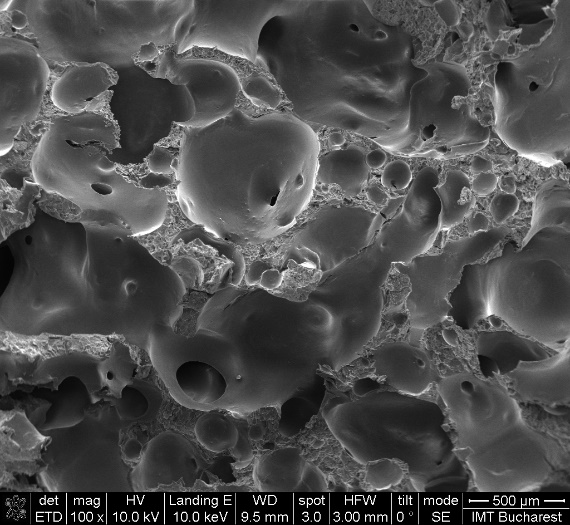 |
| 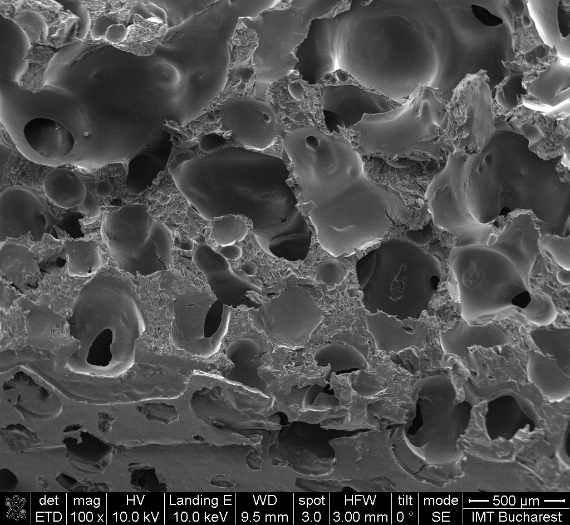 | 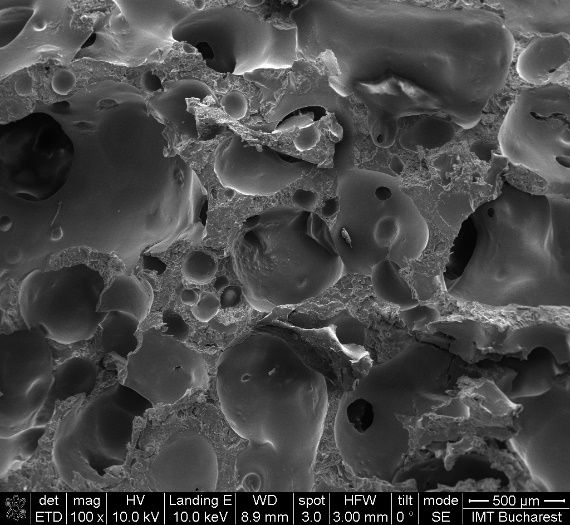 | 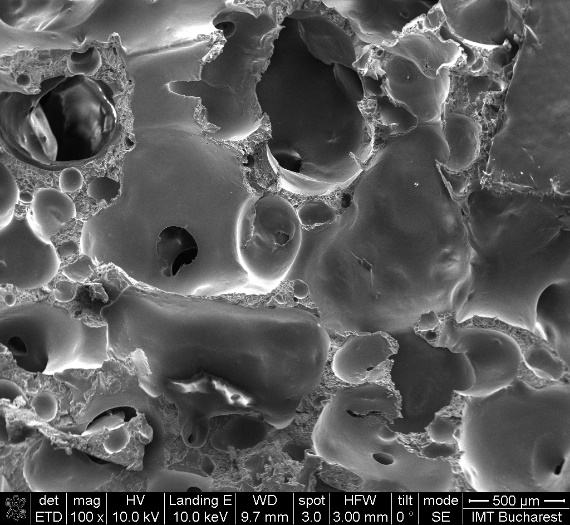 |
| **PUR-fa-6** | | |
| 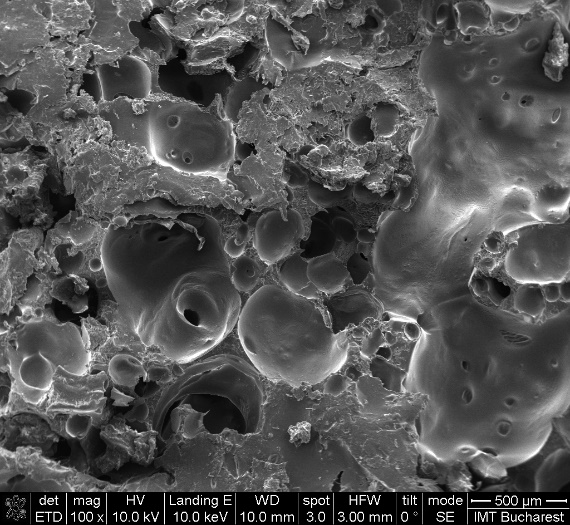 | 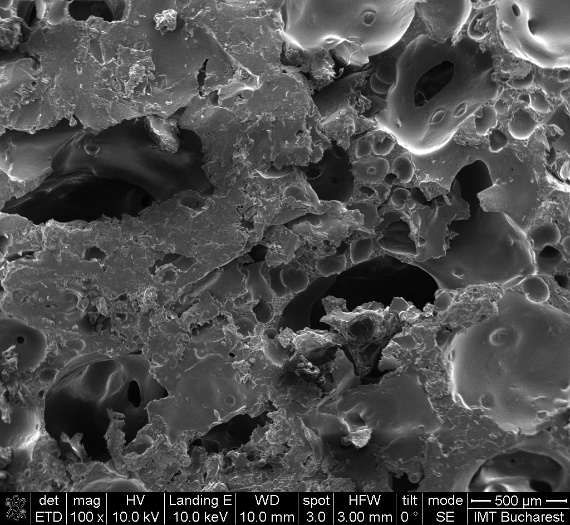 | 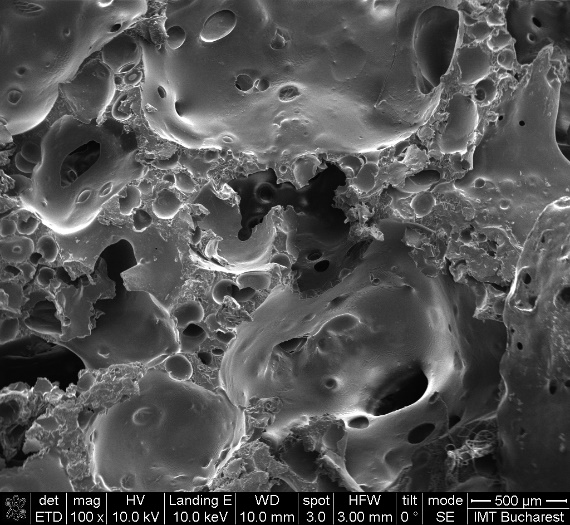 |
| 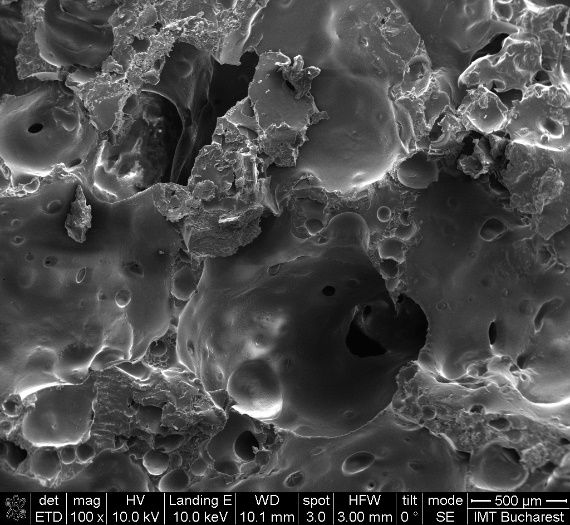 | 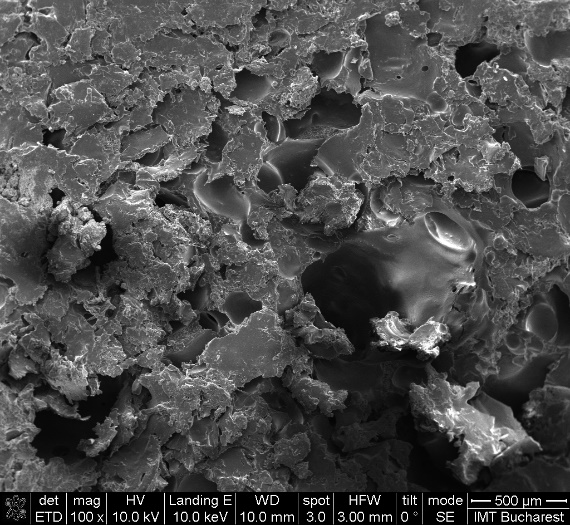 | 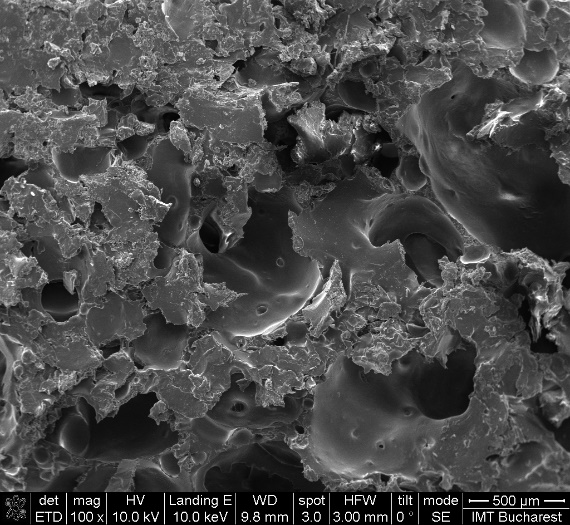 |
| **PUR-fa-8** | | |
| 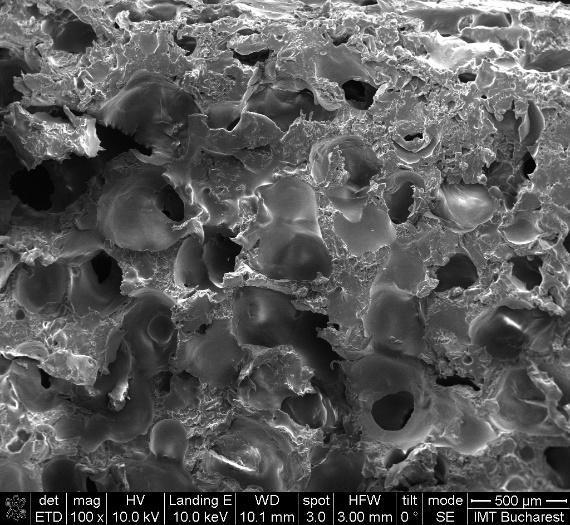 | 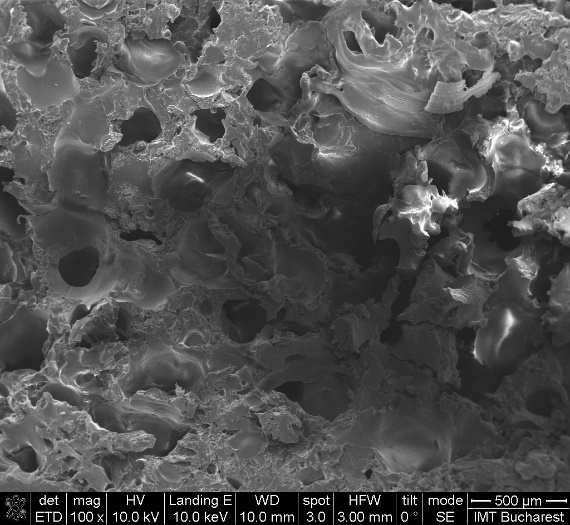 | 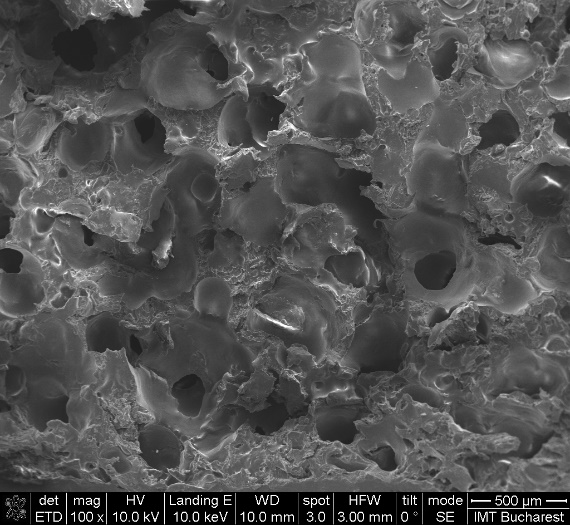 |
| 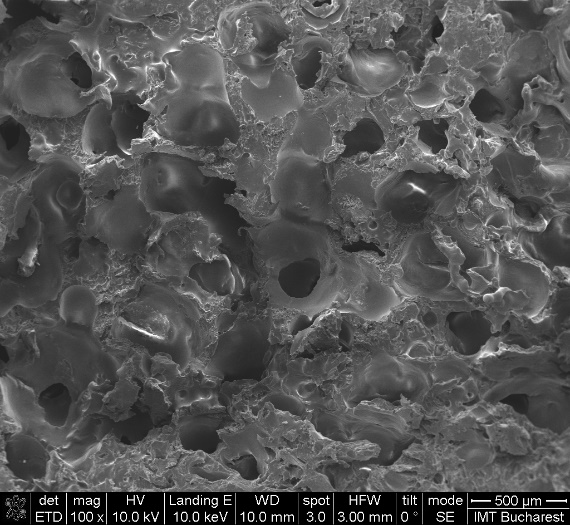 | 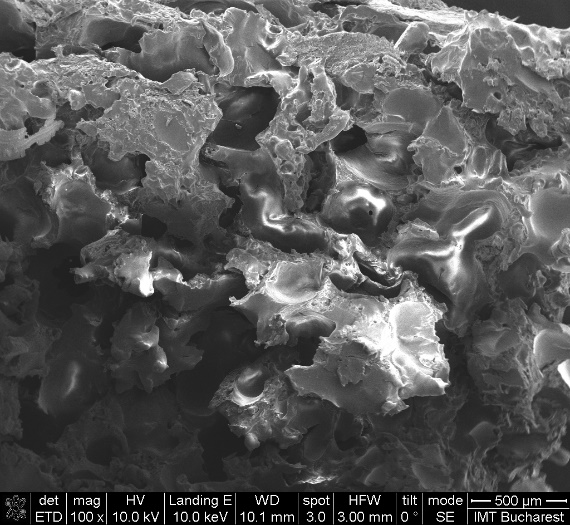 | 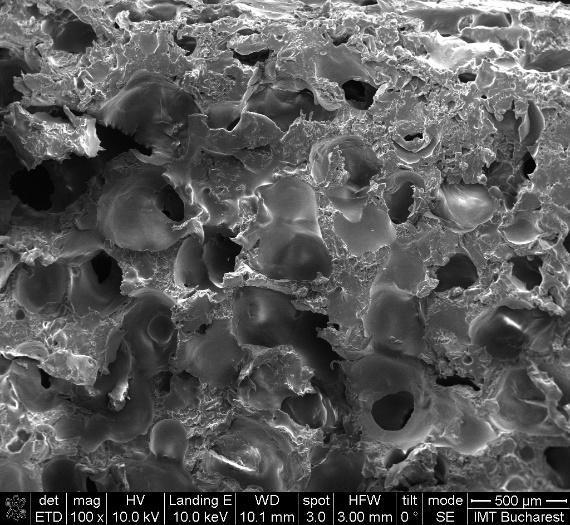 |
| **PUR-gw-4** | | |
| 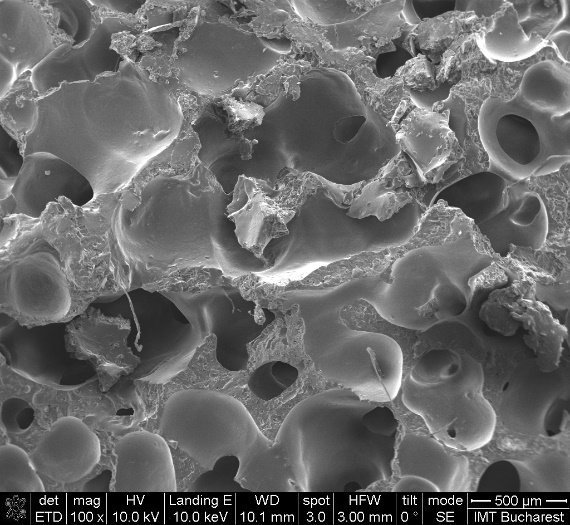 | 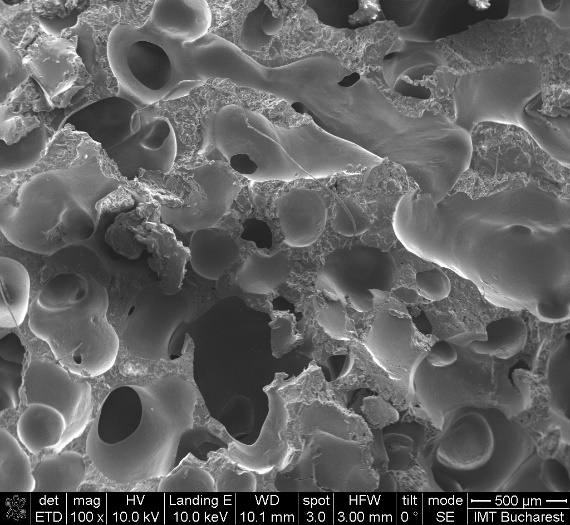 | 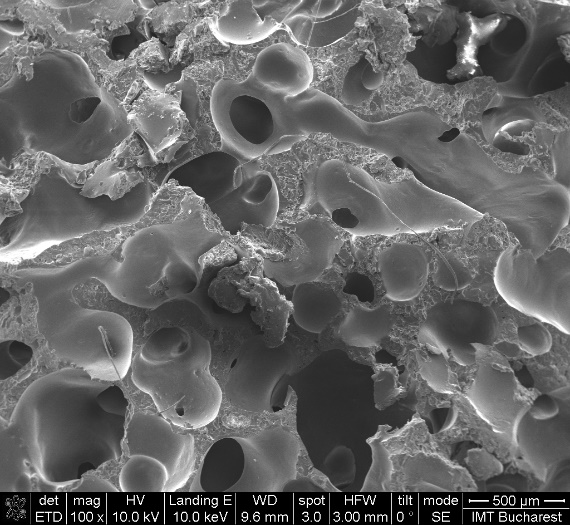 |
| 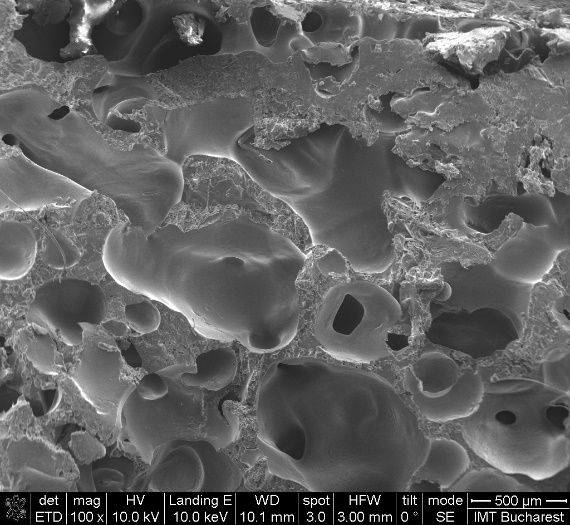 | 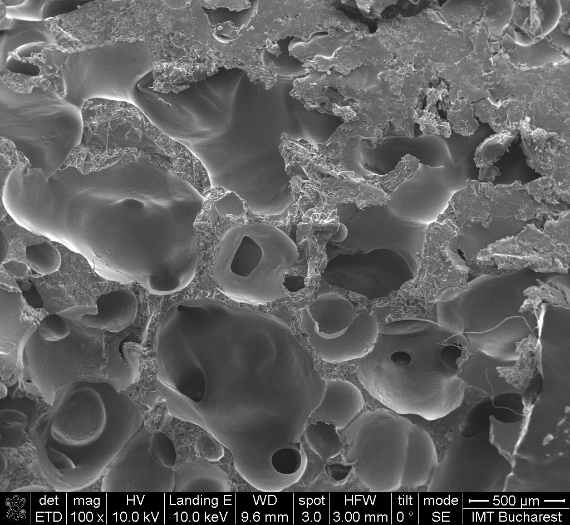 | 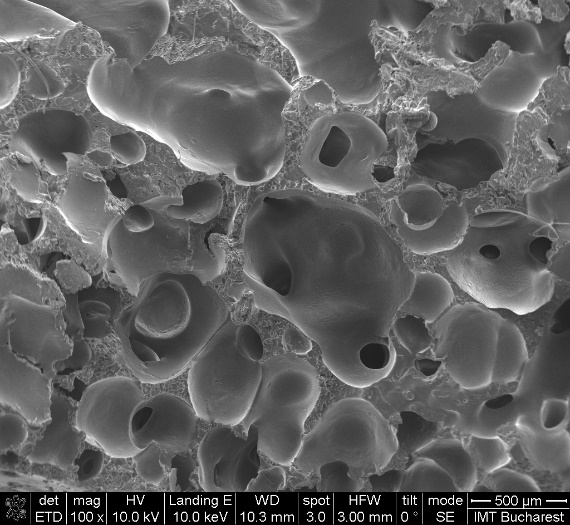 |
| **PUR-gw-6** | | |
| 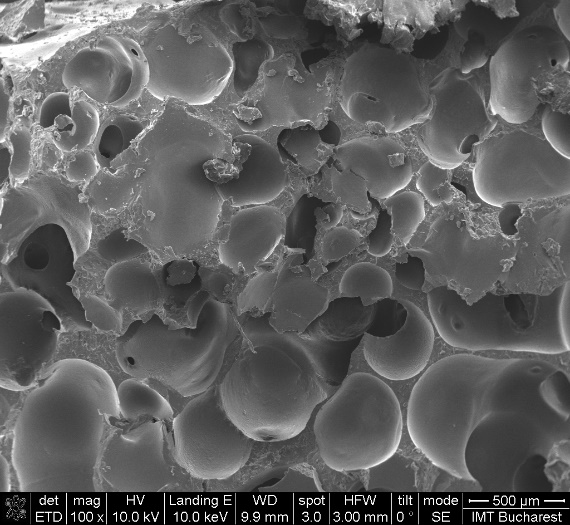 | 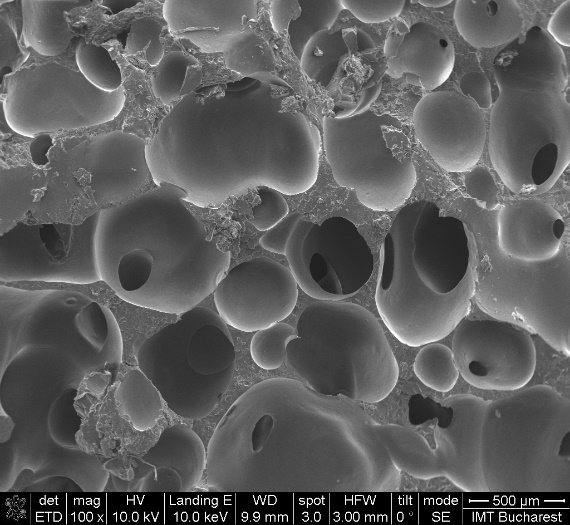 | 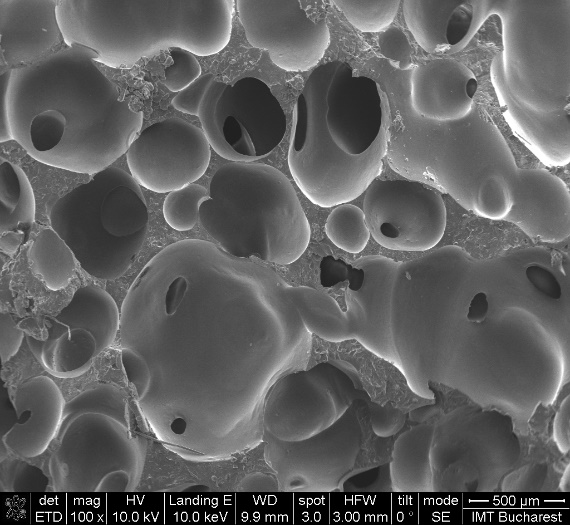 |
| 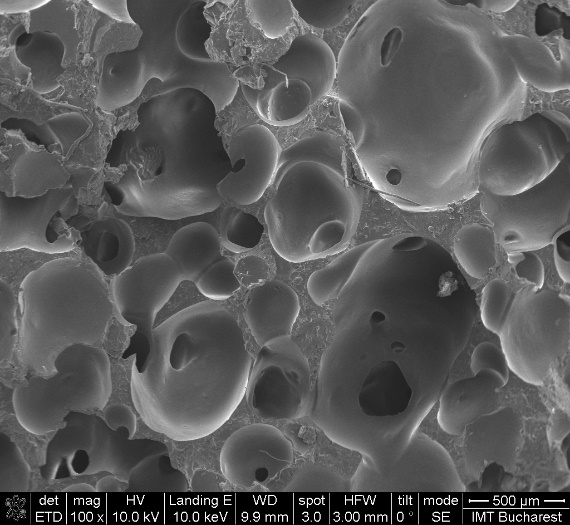 | 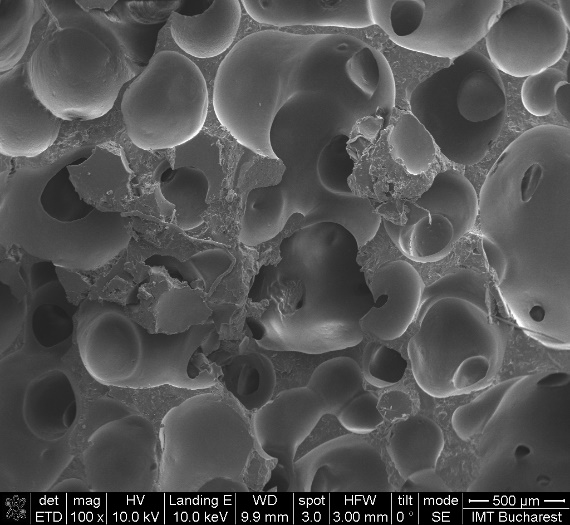 | 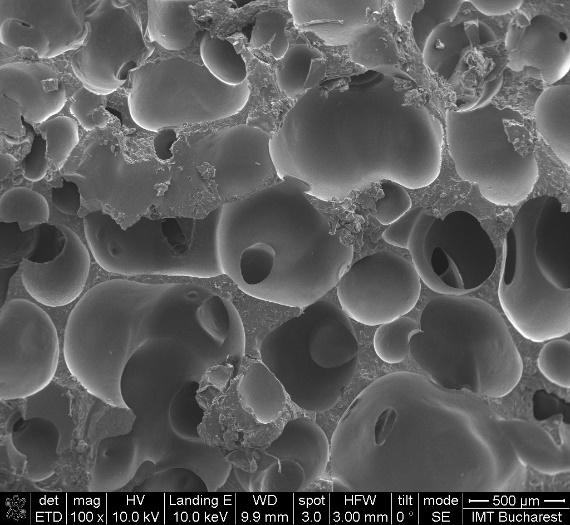 |
| **PUR-gw-8** | | |
| 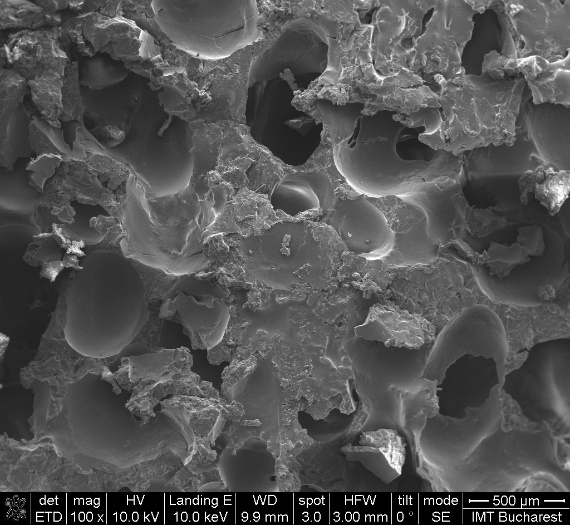 | 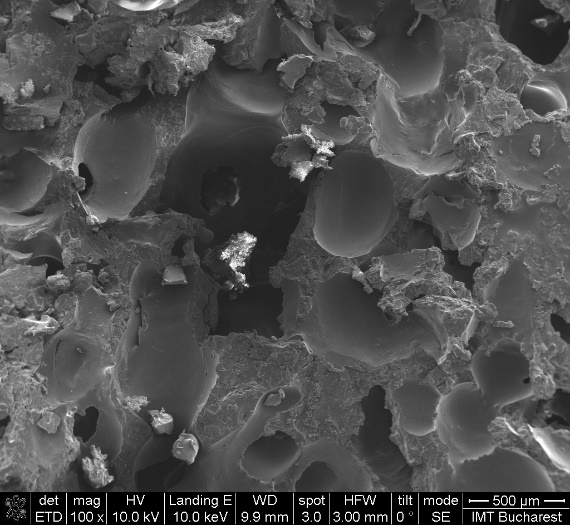 | 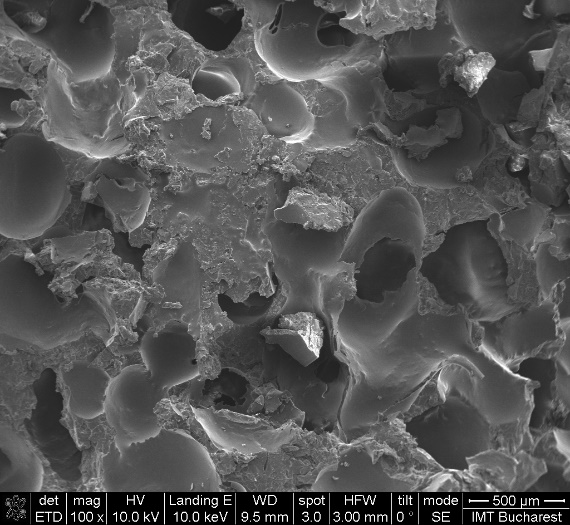 |
| 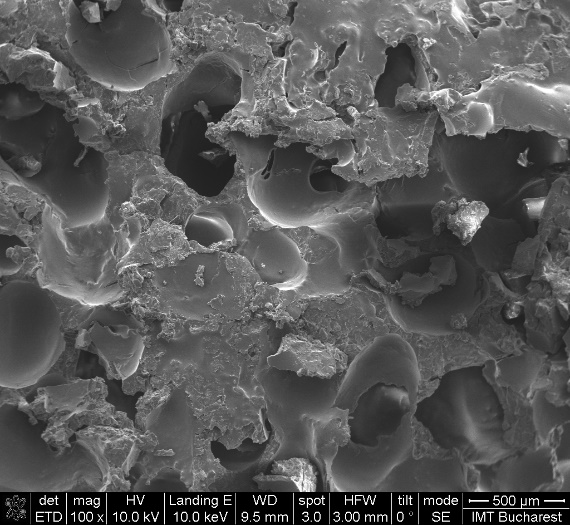 | 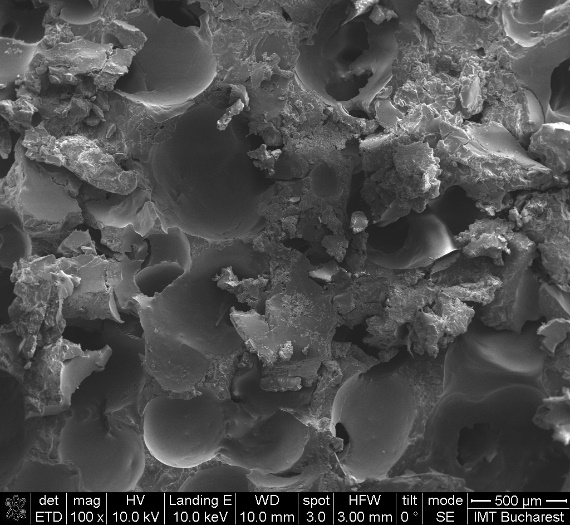 | 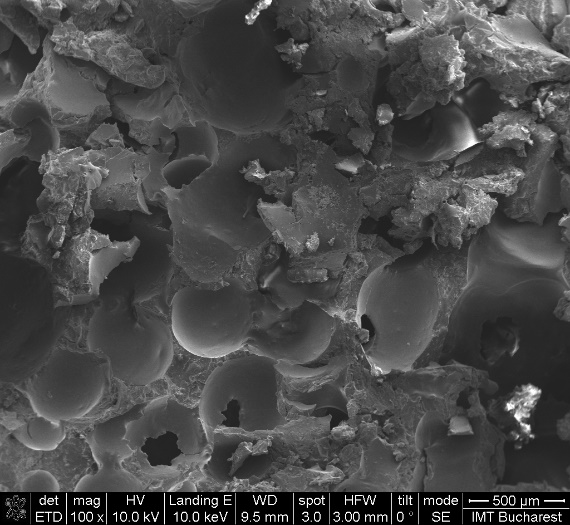 |
|  |  |  |
